# The *Aspergillus fumigatus pkcA* ^G579R^ Mutant Is Defective in the Activation of the Cell Wall Integrity Pathway but Is Dispensable for Virulence in a Neutropenic Mouse Infection Model

**DOI:** 10.1371/journal.pone.0135195

**Published:** 2015-08-21

**Authors:** Marina Campos Rocha, Krissia Franco de Godoy, Patrícia Alves de Castro, Juliana Issa Hori, Vinícius Leite Pedro Bom, Neil Andrew Brown, Anderson Ferreira da Cunha, Gustavo Henrique Goldman, Iran Malavazi

**Affiliations:** 1 Departamento de Genética e Evolução, Centro de Ciências Biológicas e da Saúde, Universidade Federal de São Carlos, São Carlos, São Paulo, Brazil; 2 Departamento de Ciências Farmacêuticas, Faculdade de Ciências Farmacêuticas de Ribeirão Preto, Universidade de São Paulo, Ribeirão Preto, São Paulo, Brazil; 3 Departamento de Farmacologia, Faculdade de Medicina de Ribeirão Preto, Universidade de São Paulo, Ribeirão Preto, São Paulo, Brazil; 4 Department of Plant Biology and Crop Science, Rothamsted Research, Harpenden, Herts, United Kingdom; 5 Laboratório Nacional de Ciência e Tecnologia do Bioetanol, Campinas, São Paulo, Brazil; The University of Wisconsin—Madison, UNITED STATES

## Abstract

*Aspergillus fumigatus* is an opportunistic human pathogen, which causes the life-threatening disease, invasive pulmonary aspergillosis. In fungi, cell wall homeostasis is controlled by the conserved Cell Wall Integrity (CWI) pathway. In *A*. *fumigatus* this signaling cascade is partially characterized, but the mechanisms by which it is activated are not fully elucidated. In this study we investigated the role of protein kinase C (PkcA) in this signaling cascade. Our results suggest that *pkcA* is an essential gene and is activated in response to cell wall stress. Subsequently, we constructed and analyzed a non-essential *A*. *fumigatus pkcA* ^G579R^ mutant, carrying a Gly579Arg substitution in the PkcA C1B regulatory domain. The *pkcA* ^G579R^ mutation has a reduced activation of the downstream Mitogen-Activated Protein Kinase, MpkA, resulting in the altered expression of genes encoding cell wall-related proteins, markers of endoplasmic reticulum stress and the unfolded protein response. Furthermore, PkcA^G579R^ is involved in the formation of proper conidial architecture and protection to oxidative damage. The *pkcA* ^G579R^ mutant elicits increased production of TNF-α and phagocytosis but it has no impact on virulence in a murine model of invasive pulmonary aspergillosis. These results highlight the importance of PkcA to the CWI pathway but also indicated that additional regulatory circuits may be involved in the biosynthesis and/or reinforcement of the *A*. *fumigatus* cell wall during infection.

## Introduction

*Aspergillus fumigatus* is a ubiquitous mold and opportunistic human pathogen that causes a number of clinical diseases including the life-threatening disease, invasive pulmonary aspergillosis (IA). Immunocompromised individuals such as those with prolonged neutropenia, recipients of hematopoietic stem-cell transplants or solid-organ transplants, and patients with advanced acquired immunodeficiency syndrome or chronic granulomatous diseases have a high incidence of IA [1]. Despite the impact of this pathogen on human health, the multitude of virulence determinants deployed by this organism and the complex interplay between them throughout the infection process are not fully understood. Such topics have been under enhanced scrutiny since the completion of the *A*. *fumigatus* genome sequence [2]. Described virulence determinants include the abundant production of highly dispersive conidia, thermo-tolerance, nutritional versatility and the secretion of secondary metabolites [3–5]. Additionally, the fungal cell wall has been shown to perform multiple roles in virulence including its structural and protective functions, its role in cell-to-cell adhesion, and finally its role in the prevention of non-self recognition by the host’s immune system [6, 7].

The fungal cell wall is a rigid and highly dynamic structure, which varies in composition among different fungal species. In *Saccharomyces cerevisiae*, the Cell Wall Integrity (CWI) pathway is the main signaling cascade governing the *de novo* synthesis of the cell wall responding to cell wall stress that arise during normal growth conditions or through environmental pressures [8]. Stimuli that activate the CWI pathway are sensed by mechanosensors located on the plasma membrane, such as Mid2 and Mtl1, and members of the Wsc protein family [9, 10]. These sensors transmit intracellular signals to the small Rho1 GTPase via the activation of two guanine nucleotide exchange factors (GEFs), Rom1-2 [8]. Once activated, Rho1p promotes the activation of Protein Kinase C (Pkc1). PKC is the apical kinase in the CWI pathway, which activates a MAPK (Mitogen-activated protein kinase) signaling cascade. The MAPK core of the *S*. *cerevisiae* CWI comprises of Bck1, the paralogues Mkk1/Mkk2 and the terminal MAPK, Mpk1 [11, 12]. The phosphorylation and activation of Mpk1 controls the function of two transcription factors, Rlm1 and SBF (Swi4/Swi6), which are responsible for regulating the expression of genes involved in cell wall biosynthesis and cell cycle control, respectively [13–15]. The PKC-CWI signaling circuit is functionally conserved among eukaryotes and has been characterized in many fungal species including *A*. *fumigatus* [16–21].

Alignment of fungal PKC sequences indicated that the proteins have a serine/threonine kinase domain and a regulatory domain comprising of the sub-domains HR1, C2 and two cysteine-rich repeats (C1A and C1B) located between the pseudosubstrate region and the fungal specific Q/A/P-rich region [21, 22]. Classical and novel PKCs contain twin C1 domains occurring in the same molecule, which are designated as C1A and C1B [23]. The C1 domain of conventional PKC enzymes was first defined due to its ability to bind phorbol esters, which are non-metabolizable structural mimics of diacylglycerol (DAG) that bind to, and activate, proteins containing C1 domains, such as conventional PKCs [24, 25]. C1 domains play a crucial role in the translocation of PKCs and other molecules from the cytosol to membranes, in response to phorbol esters, or DAG, upon receptor activation [26]. Sequence alignments and closer inspections of the yeast C1 repeats show that Pkc1p does not bind DAG [23, 27]. The same is not true for other members of the fungal family of PKCs. For example, in *Cryptococcus neoformans*, the DAG C1 binding domain of Pkc1 is required for the proper localization of laccases in the cryptococcal cell wall and for the formation of melanin [28]. In *Neurospora crassa* PKC is activated by exogenous DAG and phorbol esters, and translocated to the plasma membrane from the cytoplasm [29]. In *S*. *cerevisiae*, an interaction with the Rho1 GTPase of the CWI pathway was observed to be mediated by the Pkc1 C1 domain [27, 30]. This interaction is supported by the cell wall phenotype associated with site-specific mutations in this region of Pkc1 [31].

Previous studies have reported that *pkcA* is an essential gene in *Aspergillus nidulans* [22, 32]. In addition, a *pkcA* mutant called *calC2* was isolated in a screen for mutants showing hypersensitivity to the cell wall damaging agent, Calcofluor White (CFW), and was identified as carrying a C2537G mutation in the C1B domain [21, 33]. Besides its role in the CWI pathway, the *A*. *nidulans pkcA* gene was also described as being involved in penicillin production, morphogenesis, farnesol tolerance and cell death [21, 22, 32, 34].

The CWI pathway in *A*. *fumigatus* is partially characterized, but the mechanisms by which it is activated are not fully elucidated. In addition, the functions of other unidentified signaling circuit(s) that may coordinately operate alongside the canonical CWI pathway to promote cell wall homeostasis remain to be determined (reviewed in [35]). The present study aimed to enhance our understanding of the function of *A*. *fumigatus* CWI pathway and specifically the role of PkcA. For that purpose, we constructed and analyzed an *A*. *fumigatus pkcA* ^G579R^ mutant, carrying a Gly579Arg substitution in the C1B regulatory domain. We demonstrated that *pkcA* transcript is weakly induced in response to cell wall stress and that the *pkcA* ^G579R^ mutation compromises the activation of the downstream MAPK, MpkA, resulting in the altered expression of genes encoding cell wall-related proteins, markers of endoplasmic reticulum (ER) stress and the Unfolded Protein Response (UPR). Furthermore, *pkcA* seemed to be involved in the formation of proper conidial architecture and protection to oxidative damage in *A*. *fumigatus*.

## Materials and Methods

### Strains and culture conditions

The *A*. *fumigatus* strain *akuB* ^KU80^ [36] and the mutant strains used in this study were maintained in complete medium [YG; glucose 2% (w/w), 0.5% yeast extract (w/w), 1X trace elements) or minimal medium [MM; glucose 1% (w/w), 1x high nitrate salt solution and 1x trace elements, pH 6.5]. The composition of trace elements and high nitrate salt solution was described previously [37]. For solid complete medium (YAG) or solid minimal medium, 2% agar (w/w) was added to YG or MM, respectively. To grow the *akuB* ^KU80^ *pyrG* ^*-*^ strain, the media was supplemented with 1.2 g/l of uridine and uracil. MM+sorbitol had the same composition as MM, but contained the osmotic stabilizer D-sorbitol (1.2 M). The analysis of growth rate at different temperatures was determined by spotting 1x10^4^ conidia into the center of a 90 mm petri dish containing 20 ml of solid medium. The diameter was scored at 24 hours intervals.

To access the germination kinetics, 1x10^6^ conidia of each strain were inoculated onto glass coverslips placed within a 35 mm petri dish containing 2 ml of YG medium, which was incubated at 37°C or 45°C for 2, 4, 6 and 8 hours. After incubation, coverslips with adherent germlings were transferred to fixative solution [PBS 1X; DMSO 5% (v/v) formaldehyde 3.7% (v/v)] for 10 minutes at room temperature. Coverslips were briefly rinsed with PBS buffer, mounted and visualized in a bright field microscope. A conidiospore was counted as germinated if it possessed a germ tube, which is readily detectable as small protuberances on the spherical spore surface.

To visualize conidia and conidiophores of the *pkcA* ^G579R^ mutant compared to the wild-type strain we used a slide culture method. Briefly, 30 mL of YAG medium was poured in a 90 mm petri dish. The wide end of a 15 ml conical tube was used to punch several circles in the agar of half of the YAG plate. A sterile forceps was used to cut a line down the center o the plate and to remove the agar around the circles, so that one half of the plate is empty except for de 2–3 agar cylinders. The other half plate of YAG was maintained in order to prevent the agar cylinders form drying out in the incubator. A sterile tip was use to inoculate fresh conidia of the respective strains on the sides of the agar cylinders. A sterile coverslip was put on the top of each cylinder. Plates were incubated at 37°C during 24 to 72 hours. After the incubation, the coverslips containing the adherent hyphae and conidiophores were stained with lactophenol cotton blue solution (Fluka) and microscopically inspected.

To induce cell wall stress, 1x10^7^ conidia from wild-type and *pkcA* ^G579R^ strains were incubated in 50 ml liquid YG for 16 hours or MM for 24 hours. After incubation, 300 μg/ml of Congo Red (CR) was added to the cultures and incubated for additional 15, 30 and 60 minutes. Control was left untreated. Mycelia from each time point, pre- and post-CR exposure, were collected via vacuum filtration, immediately frozen in liquid nitrogen and stored at -80°C until used for either RNA or protein extractions.

### Construction of the *A*. *fumigatus pkcA* ^G579R^ mutant and complementing strain

The gene replacement cassette was constructed by *in vivo* recombination in *S*. *cerevisiae* as described by [38] and reported in [39]. Briefly, two fragments encompassing the *pkcA* (Afu5g11970) gene were PCR-amplified from genomic DNA of the CEA17 strain according to S1 Fig. Primers used are listed in S1 Table. The fragment amplified by the primers pkcA GC FW and pkcA 4120 REV contained the G→C transversion at the position 2044 and 250 bp of the downstream *pkcA* regulatory sequence. The 3’ *pkcA* flanking region was also PCR-amplified from genomic DNA. Primers pkcA START SC and Afu5g11970 3R contained a short homologous sequence to the multiple cloning site of the plasmid pRS426 (small letters indicated in S1 Table). The *pyrG* inserted into the gene replacement cassette was amplified from pCDA21 plasmid [40] and was used to generate a marker for prototrophy in the mutant strain. Gene replacement cassette was generated by transforming the four independent fragment along with the *Bam*HI-*Eco*RI cut pRS426, into *S*. *cerevisiae* FGSC 9721 (FY834) strain using the lithium acetate method [39]. Genomic DNA extracted from the *S*. *cerevisiae* transformant cells was used to transform *Escherichia coli* chemocompetent DH5α cells to rescue the recombined plasmid pRS426 containing the gene replacement cassette. The presence of the single mutation G2044C was confirmed by fully sequencing the *pkcA* gene within the pRS426 plasmid harboring the gene replacement cassette. The isolated plasmid was used as template to PCR-amplify the cassette using the outermost primers indicated in S1 Fig. All the PCR amplifications were performed using Phusion Hot Start II High-Fidelity DNA Polymerase (Thermo Scientific). The gene replacement cassette was transformed into *A*. *fumigatus* by using the polyethylene glycol mediated protoplast technique, according to the procedures previously described [41] but using Lallzyme MMX (Lallemand, Canada) as the lytic cocktail [39].

To complement the *pkcA* ^G579R^ strain, the *pkcA* gene plus the two 1.0 kb flanking regions was PCR amplified using the genomic DNA from the CEA17 strain as a template and primers cpkcA FW and cpkcA REV (S1 Table). Protoplasts of the *pkcA* ^G579R^ strain were transformed with the 5,871 bp PCR product and plated on media containing 300 μg/ml of CR. Several revertants, which were able to grow under these conditions, were further analyzed by PCR, using primers pkcA GC FW and Afu5g1970 3R and tested for the complementing phenotypes giving the same results. One of these were chosen and named as c*pkcA* ^G579R^.

The *pkcA* gene was also targeted for entire gene deletion. The deletion cassette was generated by *in vivo* recombination method in *S*. *cerevisiae* as described above for the *pkcA* ^G579R^ replacement cassette. Briefly, approximately 2.0 kb from the 5′-untranslated region (UTR) and 3′-UTR flanking region of the *pkcA* gene were selected for primer design (S1 Table). The primers Afu5g11970 5F and Afu5g11970 3R contained a short sequence homologous to the multiple cloning site of the pRS426 plasmid. The internal primers used for the amplification of the flanking *pkcA* regions (Afu5g11970 5R and Afu5g11970 3F, respectively for the 5’- and 3’- flanking regions) contained overhangs for *pyrG* gene. *pyrG* genes was also used as a selectable marker for uridine and uracil prototrophy and was amplified form de pCDA21 plasmid. Each fragment along with the *Bam*HI/*Eco*RI cut pRS426 was transformed into the *S*. *cerevisiae*. The deletion cassette was PCR-amplified from the recombined plasmid as described above and used for *A*. *fumigatus* transformation.

### DNA manipulation, construction of cassettes and Southern blot analysis

Southern blot analysis was used to demonstrate that the cassettes had integrated homologously at the targeted *A*. *fumigatus pkcA* locus. Genomic DNA from *A*. *fumigatus* was extracted by grinding frozen mycelia in liquid nitrogen and genomic DNA was extracted as previously described [39]. Standard techniques for manipulation of DNA were carried out as described [42]. For Southern blot analysis, *Xho*I restricted chromosomal DNA fragments were separated on 1% agarose gel and blotted onto Hybond N^+^ nylon membranes (GE Healthcare). Probe labeling for detection was performed using [α-^32^P]dCTP using the Random Primers DNA Labeling System (Life Technologies). Labeled membranes were exposed to X-ray films, which were scanned for image processing.

### Susceptibility assay to cell wall, oxidative and endosplasmic reticulum stressing agents

To monitor growth under cell wall stress 1x10^5^ conidia of each strain were inoculated onto the center of a solid YG plates supplemented with varying concentrations of caffeine (CAF), Calcofluor White (CFW), anidulafungin [AF; (Ecalta, Pfizer)], CR, Sodium Dodecyl Sulfate (SDS) and ethylenediaminetetraacetic acid (EDTA), as specified in the results section. Alternatively, to assess sensitivity to nikkomycin Z (NKZ), and fluconazole (FLUC), 10 fold serial dilutions of conidia from the wild-type and mutant strains were used. The plates were incubated for 2–3 days at 37°C, and the extent of vegetative growth was used as a relative indicator of sensitivity. For the experiments using solid MM supplemented with 1.2 M sorbitol, serial dilutions of conidia ranging from 1x10^6^ to 1x10^3^ were spotted onto agar plates. For the evaluation of the oxidative stress tolerance, 1x10^5^ conidia were inoculated in 24-well plates containing 1 ml of liquid MM and varying concentration of menadione or paraquat, as specified in the results section. The sensitivity to reactive oxygen species (ROS) generated by diamide and H_2_O_2_ was tested by an inhibition zone assay in MM agar plates, as described by [19]. ER stress in the presence of DTT (dithiothreitol) was likewise tested in liquid YG culture, while brefeldin A (BFA) and tunicamycin (TM) were tested in solid media, as previously described for *A*. *fumigatus* [43].

### Survival in the presence of chelerythrine

To assess if the PKC inhibitor, chelerythrine, was fungicidal to the *pkcA* mutant, 2x10^3^ conidia of the wild-type, *pkcA* ^G579R^ and complementing strains were inoculated in 200 μl of liquid MM containing different concentrations of chelerythrine in 96 well polystyrene plates. Cells were grown for 24 hours at 30°C. After this incubation plates were centrifuged (3,600 g) to remove the media and washed three times with pre-warmed fresh MM. 200 μl of fresh MM were added to the plates and the cells were allow to recover for additional 24 hours at 30°C. After incubation, plates were centrifuged again and incubated with 200 μl of MTT (Thiazolyl Blue Tetrazolium Bromide) solution (10 mg/ml in PBS) for three hours in the dark at 37°C to permit the reduction of MTT and the formation of formanzan salt. Plates were centrifuged (3,600 g for 10 minutes). The content of each well was removed and 100 μl of isopropanol containing 5% (v/v) 1 M HCl was added to dissolve the formanzan crystals. Plates were incubated overnight at room temperature in the dark. The optical density was measured spectrophotometrically with a microtiter plate reader at 570 nm (Biorad). The mean MTT determinations were obtained from 12 replicates per plate, with five independent experiments. The results were expressed as mean ± SD and were considered statistically different with a *p*-value ≤ 0.05, as determined by the Student’s T test using Graph-Pad Prism software.

### Antifungal susceptibility (Etest diffusion assay)

E-test strips (Probac) were used according to the manufacturer’s instructions to determine *A*. *fumigatus* susceptibility to caspofungin, voriconazole and amphotericin B. Briefly, conidial suspensions were prepared in sterile MilliQ water and counted using the Neubauer chamber. 1x10^6^ conidia were used to inoculate 90 mm plates of RPMI 1640 agar (Sigma R1383) buffered with MOPS (pH 7.0). 100 μl of conidial suspension was spread evenly onto the surface of the agar plates using a wetted swab. Plates were allowed to dry for 20 min before Etest strips were applied. The plates were incubated at 37°C and analyzed after 24 and 48 hours before being photographed. The Minimal Inhibitory Concentration (MIC) was defined as the lowest drug concentrations at which the border of the elliptical inhibition zone intercepted the scale on the antifungal strip.

### RNA extraction, real time RT-PCR procedures and analysis of *hacA* splicing by RT-PCR

Genes that have well-known or putative functions in cell biosynthesis and reinforcement were chosen for gene expression studies by using real time RT-PCR. Mycelia was disrupted by grinding in liquid nitrogen with a pestle and mortar and the total RNA was extracted using the Trizol reagent (Life Technologies) according to the manufacturer’s protocol. Samples were treated with Turbo DNase I treatment (Life Technologies) to remove genomic DNA. The DNAse treatment was validated by real time PCR using *A*. *fumigatus* β-tubulin (*tubA*) primers using the DNAse-treated RNA as template in the reactions. DNAse-treated RNA quality was confirmed by denaturing agarose gel (2.2 M formaldehyde; 1.2% (wt/vol) agarose) stained with ethidium bromide, and visualized under UV light to evaluate the presence of intact 25S and 18S rRNA bands. RNA concentration and quality were measured with a nanophotometer (NanoVue, GE HealthCare). RNA integrity was assessed using a 2100 Bioanalyzer (Agilent Technologies). A total of 2 μg of DNAse-treated total RNA from each *A*. *fumigatus* strain was reverse transcribed with High Capacity cDNA Reverse Transcription kit (Life Technologies) using oligo dTV and random primers blend. Real-time RT-PCR was conducted using Power Sybr Green PCR Master Mix (Life Technologies). Primers for the individual cell wall biosynthesis genes were designed using Primer Express 3.0 software (Life Technologies) and are listed in S2 Table. Real time RT-PCR was performed in duplicate with three independent biological samples in a StepOne Plus Real Time PCR System (Life Technologies). The concentration of each primer pair was optimized prior to the efficiency curve reaction. Only primers having amplification efficiency ranging from 95%-105% were used according to reference [44]. Non-template controls (NTC) were used to confirm elimination of contaminating DNA in every run. Melt curve analysis was performed after the PCR was complete to confirm the absence of non-specific amplification products. The fold change in mRNA abundance was calculated using 2^−ΔΔCt^ [45] and all values were normalized to the expression of the *A*. *fumigatus* β-tubulin (*tubA*, encoding β-2 tubulin subunit; Afu1g10910) gene.

The *hacA* mRNA splicing analysis was carried out according to the protocol described previously for *A*. *fumigatus* [46] with minor modifications. Briefly, RNA was extracted and reverse transcribed exactly as described above and the Afu hacA (u-i) FW and Afu hacA (u-i) REV (S2 Table) were used in the RT-PCR reaction using the first-strand cDNA as a template. This primer pair flanks the unconventional 20 nucleotide intron and yield fragments of 120 bp or 100 bp for the *hacA* uninduced (non-spliced) and *hacA* induced (spliced) transcripts. The cycling conditions were: 98°C, 10 s; 25 cycles of 98°C, 1 s; 52°C, 5 s; 72°C, 10 s; 72°C, 1 min, using Phusion Flash High-Fidelity PCR Master Mix (Thermo Scientifc). Amplicons were load onto a 12% acrylamide/7M urea gel in 1X TBE after heating the samples (95°C; 5 minutes) in RNA loading buffer. The PCR products were stained with ethidium bromide for visualization and image capture. The images generated were subjected to densitometric analysis using the ImageJ software [47]. The cDNA loading for each sample was normalized by *tubA* amplified with primers tubA FW e tubA REV, described in S2 Table.

### Protoplast counting

To assess the ability of the *pkcA* ^G579R^ strain to generate protoplasts under standard conditions containing cell wall-degrading enzymes, 2x10^6^ conidia from each strain were inoculated in 50 ml liquid YG and incubated for 16 hours at 37°C (180 rpm). Cells were washed twice with sterile MilliQ water and 100 mg of mycelium wet weight were incubated in 50 ml of a osmotic stabilized protoplasting solution [(0.4 M ammonium sulfate; 50 mM citric acid pH 6.0; yeast extract 0,5% (w/v), sucrose 1% (w/v)] according to reference [39] containing 0,3% of Lallzyme MMX as lytic cocktail (β-glucanase and pectinase blend sourced from *Trichoderma sp*. and *A*. *niger*) and 400 mg of BSA at 30°C (90 rpm). The protoplasts yield was analyzed using Neubauer chamber after 0, 4 and 6 hours of incubation.

### Staining for dectin-1 and chitin

The staining was performed as described previously [48, 49]. Briefly, *A*. *fumigatus* conidia were grown for 8 hours at 37°C in liquid MM, UV-irradiated, blocked using blocking solution (goat serum 2%, BSA 1%, 0.1% Triton X-100, 0.05% Tween 20, 0.05% NAF and 0.01 M PBS) for 1 hour at room temperature, and stained with conditioned medium containing 1 μg/ml of s-dectin-hFc followed by DyLight 594-conjugated, goat anti-human IgG1 [50]. For chitin staining, UV-irradiated germlings were treated with CFW 2 μg/ml for 5 minutes. After washing, stained cells were visualized under identical imaging conditions for parallel comparison using a Zeiss Observer Z1 fluorescence microscope. Staining was quantified as the averaged amount of staining to the total fungal area using ImageJ software.

### Biofilm formation assay

The quantification of the initial stages of biofilm formation in *A*. *fumigatus* was carried out as described by [51]. Briefly 2x10^4^ conidia were inoculated into 200 μL YG media in 96- well polystyrene plates and allowed to grow for 24 hours at 37°C. After incubation, the media was removed and the adhered mycelia were washed four times with sterile PBS. 150 μl of a 0.5% (w/v) crystal violet solution was added to each well for 5 minutes to stain the residual mycelia. Excess stain was gently removed under running water. The residual biofilm was destained with 200 μl 95% ethanol per well, overnight at room temperature. Biofilm density was measured by determining the absorbance of the destaining solution at 570 nm (BioRad).

### Rodlet layer extraction

The hydrophobins were extracted from the dormant spore surface by incubating dry conidia with 48% hydrofluoric acid (HF) for 72 h at 4°C according to reference [52]. Briefly, the contents were centrifuged (9,000 g for 10 min) and the supernatant obtained was dried under N_2_. The dried material was reconstituted in milliQ H_2_O and an aliquot was subjected to 15% SDS-PAGE gel. Bands corresponding to the 16 kDa and 14.5 kDa RodA protein were visualized by silver nitrate staining following standard protocols. The amount of conidia of each strain subjected to HF extraction was further validated by CFU (colony forming unit) counting onto YG solid medium.

### Protein extraction and western blot analysis of phosphorylated MpkA

To assess the phosphorylation status of MpkA, freshly harvested conidia (1x10^7^) of the wild-type and *pkcA* ^G579R^ strains were inoculated in 50 ml liquid YG medium at 37°C for 16 hours (180 rpm). After incubation, 300 μg/ml of CR was added to the cultures and incubated for an additional 30, 60 and 120 minutes. Control was left untreated. The phosphorylation of MpkA upon pharmacological inhibition of PkcA by chelerythrine was performed by growing the wild-type and *pkcA* ^G579R^ as mentioned above. Then chelerythrine (25 μM) was added or not (control) for 120 minutes.

Mycelia were ground in liquid nitrogen with pestle and mortar. For protein extraction, 0.5 ml lysis buffer described in reference [20] containing 10% (v/v) glycerol, 50 mM Tris–HCl pH 7.5, 1% (v/v) Triton X-100, 150 mM NaCl, 0.1% (w/v) SDS, 5 mM EDTA, 50 mM NaF, 5 mM sodium pyrophosphate, 50 mM β-glycerophosphate, 5 mM sodium orthovanadate, 1 mM PMSF, and 1X Complete Mini protease inhibitor (Roche Applied Science) was added to the ground mycelium. Extracts were centrifuged at 20,000 g for 40 minutes at 4°C. The supernatants were collected and the protein concentrations were determined using the Hartree method [53]. 50 μg of protein from each sample were resolved in a 12% (w/v) SDS–PAGE and transferred to polyvinylidene difluoride (PVDF) membranes (BioRad). The phosphorylation of the MAP kinase MpkA, was examined using anti-phospho p44/42 and anti p44/42 MAPK antibody (9101 and 9102, respectively; Cell Signaling Technologies) following the manufacturer’s instructions using a 1:1000 dilution in TBST buffer (137 mM NaCl, 20 mM Tris, 0.1% Tween-20) containing 5% BSA and 16 hours incubation at 4°C. Primary antibody was detected using an HRP-conjugated secondary antibody raised in rabbit (A0545; Sigma). Anti γ-tubulin (yN-20; Santa Cruz Biotechnology) were used as loading control in the experiments. Incubation was performed in a 1:2000 dilution in TBST containing 3% skimmed milk and incubated in a rocking platform for 16 hours at 4°C. Anti γ-tubulin antibodies were detected by peroxidase (HRP)-conjugated second antibody (Sigma). Chemoluminescent detection was achieved by using ECL Prime Western Blot detection kit (GE HealthCare). Images were generated by exposing the membranes to the ChemiDoc XRS gel imaging system (BioRad). The images were subjected to densitometric analysis using ImageJ software [47].

### BMDMs preparation, phagocytosis index and determination of TNF-α levels

For cytokine quantification and phagocytosis index determination, Bone Marrow Derived Macrophages (BMDMs) from C57BL/6 mice were prepared as 360 previously described [54]. Briefly, bone marrow cells from femurs of adult mice were cultured for 6 days in RPMI 1640, containing 20% fetal bovine serum (FBS) and 30% L-929 cell conditioned media (LCCM).

The phagocytic assay was performed according to [55]. Briefly, in a 24-well plate containing one 15 mm diameter coverslip per well, 2x10^4^ macrophages were incubated with 1 ml of RPMI-FBS at 37°C with 5% CO_2_ for 1 hour. Next, the cells were washed with 1 ml of assay medium to remove non-adherent cells. In each well, 1 ml of RPMI-FBS containing 1x10^5^ conidia (1:5 macrophage/conidia ratio) was added. The samples were incubated at 37°C with 5% CO_2_ for 80 min, then the supernatant was removed and 500 μl of 3.7% formaldehyde–PBS was added. After 15 min, the samples were washed with 1 ml of ultrapure water and incubated for additional 20 min with 495 μl of water and 5 μl of CFW (10 mg/ml). The samples were washed and mounted on slides with 50% glycerol. A Zeiss Observer Z1 fluorescence microscope was used to assess the percentage of phagocytized spores. Since macrophage cells were not permeable, only internalized conidia remained unstained by CFW. At least 100 conidia were counted per sample, and a phagocytosis index was calculated.

For TNF-α, macrophages (5x10^5^) were plated in 48-well plates for 16 h at 37°C, 5% CO_2_ in RPMI 140 media containing 10% FBS and 5% of LCCM. For fungal infection, strains were cultured for 18 hours up to a hyphal stage at a density of 2x10^4^ per well, UV-irradiated and used to stimulate the BMDMs. The cells were centrifuged to synchronize the infection and allowed to infect for 18 h. The supernatant was collected and the cytokine was measured by enzyme-linked immunosorbent assay (ELISA) with a mouse TNF-α kit (R&D Quantikine ELISA) according to the manufacturer's instructions. For positive control, it was used 1μg/ml of LPS from *E*. *coli* (Sigma).

### Animal model of invasive pulmonary aspergillosis and ethics statement

Virulence of *A*. *fumigatus* strains was analyzed using a murine model for invasive aspergillosis, as detailed described by Dinamarco *et al*. [56]. Briefly, outbreed female mice (BALB/c strain; body weight, 20 to 22 g) were housed in vented cages containing 5 animals. Mice were immunosuppressed with cyclophosphamide at 150 mg/kg of body weight administered intraperitoneally on days -4, -1, and 2 prior to and postinfection. Hydrocortisonacetate (200 mg/kg) was injected subcutaneously on day -3. *A*. *fumigatus* conidia used for inoculation were grown on YAG medium for 2 days prior to infection. Conidia were freshly harvested in PBS and filtered using Miracloth (Calbiochem). Conidial suspensions were spun for 5 min at 3,000 x g, washed three times with PBS, counted using a hemocytometer, and resuspended at a concentration of 2.5x10^6^ conidia/ml. Viable counts of the administered inocula were determined, following serial dilution, by plating on YAG medium, and the conidia were grown at 37°C. Mice were anesthetized by halothane inhalation and infected by intranasal instillation of 5.0x10^4^ conidia in 20 μl of PBS. As a negative control, a group of 5 mice received sterile PBS only. Mice were weighed every 24 h from the day of infection and visually inspected twice daily. In the majority of cases, the endpoint for survival experimentation was identified when a 20% reduction in body weight was recorded, at which time the mice were sacrificed. The statistical significance of comparative survival values was calculated using log rank analysis and the Prism statistical analysis package.

This study and the protocols herein described involving animal care were approved by the Local Ethics Committee for Animal Experiments from the Federal University of São Carlos—UFSCar (Permit Number: Protocolo CEEA n° 062/2009). All animals were housed in groups of five within individually ventilated cages and were cared for in strict accordance with the principles outlined by the Brazilian College of Animal Experimentation (Sociedade Brasileira de Ciência em Animais de Laboratório–SBCAL (formerly COBEA—Colégio Brasileiro de Experimentação Animal). All efforts were made to minimize suffering. Animals were clinically monitored at least twice daily and humanely sacrificed if moribund (defined by lethargy, dyspnea, hypothermia and weight loss). All stressed animals were sacrificed by cervical dislocation.

## Results

### Construction of the *pkcA* ^G579R^ mutant strain

A BLASTp search of the *A*. *fumigatus* genome identified a single uncharacterized open reading frame (Afu5g11970) as the putative homologue of the *S*. *cerevisiae* PKC1 and the *A*. *nidulans* PkcA, which was hence named PkcA, to be consistent with the previous nomenclature for this gene in *A*. *nidulans* [22]. The *A*. *fumigatus* PkcA sequence is 1,106 amino acids and the location of each domain was reported previously [21, 22]. The genome of *A*. *fumigatus* contains a single PKC orthologue, which is similar to other fungi except for *Schizosaccharomyces pombe* and *Sporothrix schenckii* where an additional PKC copy is present [57, 58]. PkcA protein sequences from *A*. *nidulans* and *A*. *fumigatus* displayed 84% amino acid identity and 88% protein sequence similarity (e-value 0.0), while *S*. *cerevisiae* Pkc1p and *A*. *fumigatus* PkcA displayed 66% amino acid identity and 82% protein sequence similarity (e-value 2.1e-255).

As an initial approach to functionally characterize the *A*. *fumigatus pkcA* gene, we took advantage of a well-defined mutation in *A*. *nidulans* gene encoding PkcA, named *calC2*, and created an *A*. *fumigatus* mutant carrying the same mutation within the *pkcA* gene sequence. This mutation is located at nucleotide position 2044 and consists of a G to C transversion inside the cysteine-rich C1B regulatory domain. This mutation is located beside the C-terminal limit of the C1B domain and introduces a charged arginine residue replacing the original neutral glycine, which is highly conserved among fungal and mammals PKCs [21].

The gene replacement cassette for the generation of the *pkcA* ^G579R^ mutant strain was obtained using an *in vivo S*. *cerevisiae* fusion-based approach [39] and consisted of a cassette containing the mutated *pkcA* sequence followed by 250 bp of the endogenous terminator region (S1 Fig). The introduction of the G2044C point mutation was confirmed by sequencing the entire *pkcA* gene from the cassette. No additional mutations were observed (data not shown). Replacement of *pkcA* locus by the substitution cassette was rigorously confirmed by diagnostic PCR (data not shown). Replacement occurred in several transformants, which were further confirmed by Southern blotting analysis of the *Xho*I-digested genomic DNA of the mutant indicating a correct and single integration of the replacement cassette. One of these transformants was selected for further phenotypic characterization and was named as *pkcA* ^G579R^ (S1 Fig). The mutant allele was also complemented with the corresponding wild-type gene (c*pkcA* ^G579R^ strain) aiming to confirm the occurrence of possible secondary mutations during the construction of the deletion strain. Complementation of the *pkcA* gene in the *pkcA* ^G579R^ mutant background was confirmed by PCR (S1 Fig). Complemented strain was indistinguishable from the wild-type strain.

### The *pkcA* gene is essential for viability and is required for vegetative growth in *A*. *fumigatus*

**Fig 1:**
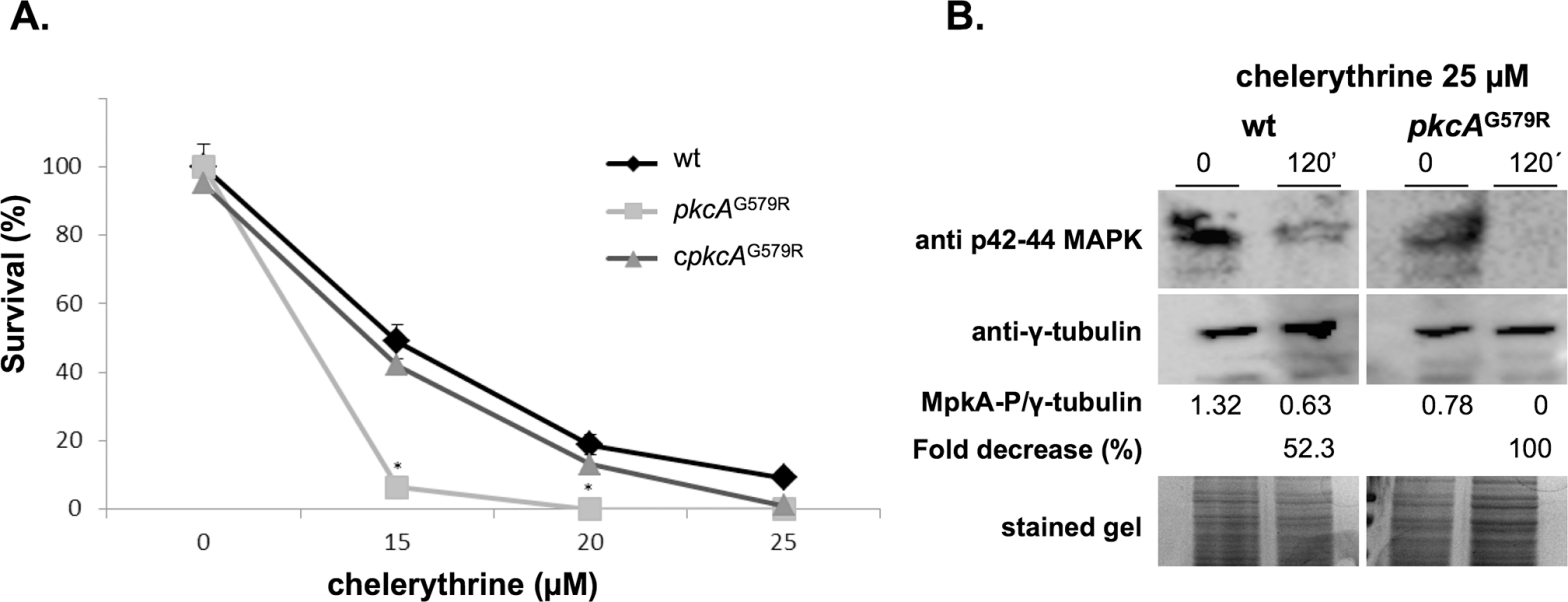
Chelerythrine treatment leads to a fungicidal effect of *A*. *fumigatus pkcA* ^G579R^ mutant. (A) 2x10^3^ conidia from each strain were grown for 24 hours in liquid MM at 30°C in the presence of the indicated concentration of chelerythrine. After growth, cells were centrifuged, washed in pre-warmed medium and allow to recover in fresh MM medium for an additional 24 hours. After this time, survival was determined via the MTT assay comparing the formanzan salt formation at each time point in comparison to the untreated control. * p ≤ 0.01. (B) Western blot for MpkA phosphorylation. Wild-type and *pkcA* ^G579R^ strains were grown for 16 h in YG at 37°C and then chelerythrine (25 μM) was added, or not (control), to the medium for 120 minutes. Anti-phospho-p44/42 MAPK antibody was used to detect the phosphorylation of MpkA. The γ-tubulin antibody and Coomassie Brilliant Blue stained gels were used as loading sample controls. Signal intensity was quantified using the ImageJ software by dividing the intensity of phosphorylatred MpkA/γ-tubulin and expressed as arbitrary units.

*A*. *fumigatus* PkcA protein domain organization resembles the structure of novel PKC’s, as reported for *A*. *nidulans* [21, 22]. Previously, the *pkcA* orthologue in *A*. *nidulans* was shown to be an essential gene [22, 32, 59]. Consequently, we began our examination of the *A*. *fumigatus* CWI pathway by attempting to generate a *pkcA* null mutant. In spite of many attempts, we were unable to obtain any transformants for the *pkcA* gene deletion, even using Δ*akuB* ^KU80^ that is deficient for non-homologous end joining and favors homologous recombination [36]. These results suggested that *pkcA* in *A*. *fumigatus* is also essential for viability. As an attempt to further investigate the effects of *pkcA* inactivation in *A*. *fumigatus*, we looked for PKC pharmacological inhibitors that acted directly on the catalytic kinase domain, which is separate from the C1B motif located in the PKC regulatory domain [22]. Clerythrine (CHE) is described as a potent inhibitor of PKC interacting with the catalytic domain of PKC and has been shown to inhibit *S*. *cerevisiae* growth [60, 61]. So, we reasoned that it would be possible to observe a synthetic lethality effect derived from the partial loss of function of *pkcA* in the *pkcA* ^G579R^ mutant in the presence of this drug. The MIC determination for CHE in *A*. *fumigatus* performed in YAG medium according to the Clinical and Laboratory Standards Institute (CLSI) M38-A2 protocol was 20 μM (data not shown). Accordingly, we exposed conidia form wild-type, mutant and complemented strains to different concentrations of CHE below and above the MIC for 24 hours and then measured the ability of the cells to resume growth in fresh media. Viability was determined after 24 hours of recovery and quantified in a MTT assay. Survival of the mutant strain was reduced in comparison to the wild-type and complemented strains at lower drug concentration (Fig 1A). The wild-type and complementing strains showed about 20% survival at the MIC concentration, while the mutant was not viable (0%). In order to demonstrate that this was due to inhibition of *A*. *fumigatus* PkcA by CHE, MpkA phosphorylation was examined by Western blot assay. The results show that there was no phosphorylation of MpkA after 120 minutes of CHE exposure in the *pkcA* ^G579R^ strain and a reduction of about 52.3% in the wild-type strain (Fig 1B). These results indicate that the G579R mutation in C1B domain of PkcA combined with the inhibition of the catalytic site of the protein lead to a fungicidal effect.

**Fig 2:**
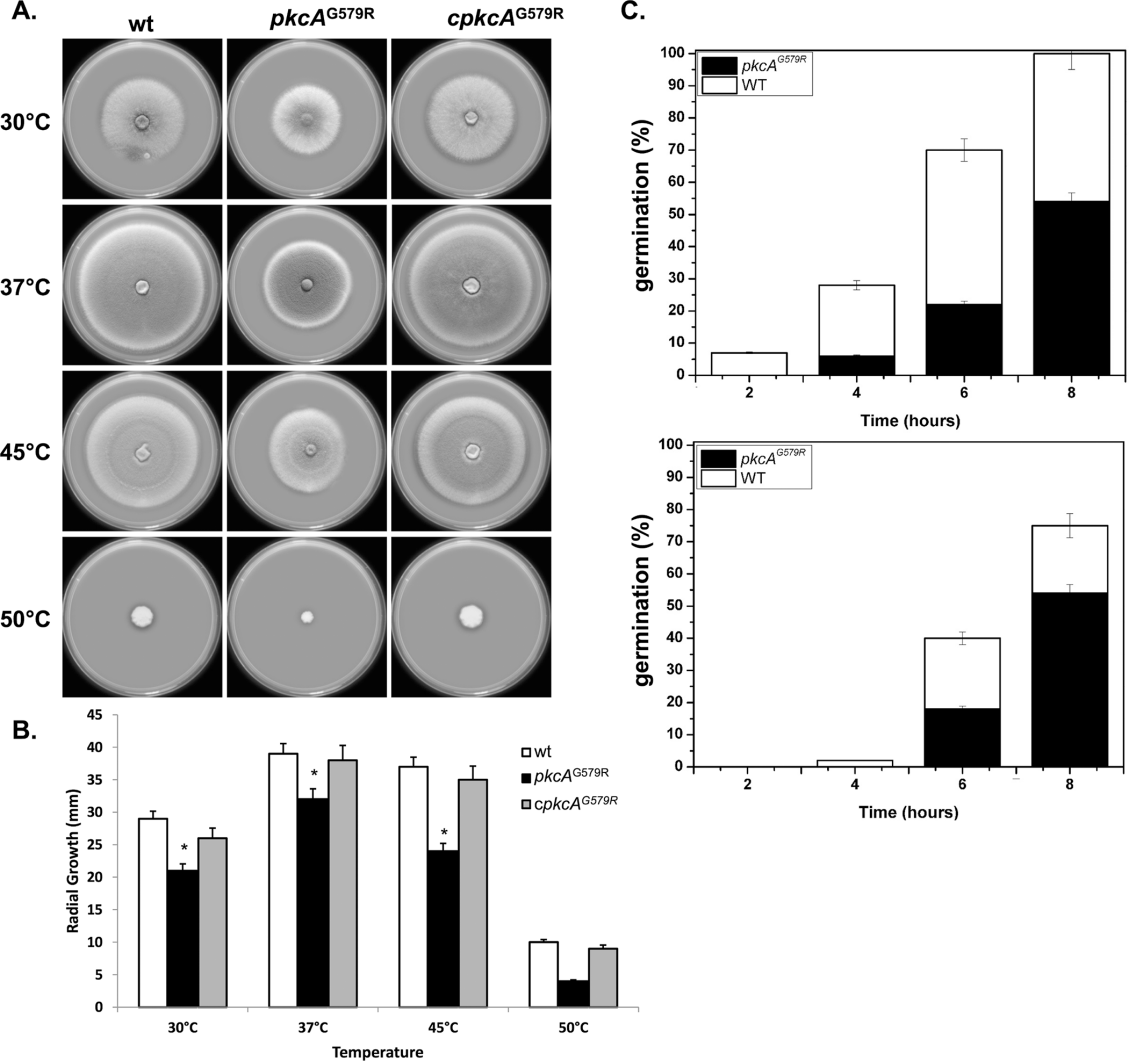
PkcA contributes to thermotolerant growth and germination in *A*. *fumigatus*. 1x10^4^ conidia of each strain were inoculated onto the center of a YAG medium and radial growth was measured after 3 days at the indicated temperatures (A-B). 1x10^6^ conidia of each strain were inoculated in 2 ml liquid YG culture and incubated at 37°C and 45°C during 2, 4, 6 and 8 hours before the percentage of germination was evaluated (C). Experiments were performed in triplicate and the results are the mean ± standard deviation. * Statistically significant by Student’s T-test (p ≤ 0.01).

An initial phenotypic analysis of *A*. *fumigatus pkcA* ^G579R^ mutant strain displayed decreased radial hyphal growth at all temperatures tested and this difference was more evident at 45°C and 50°C. The radial growth rate of the complemented strain was similar to the wild-type strain, but not the *pkcA* ^G579R^ mutant, in both complete (Fig 2A) and minimal medium (data not shown). At 37°C the radial growth rate of the *pkcA* ^G579R^ mutant was approximately 10% less than the wild-type and complemented strains, while at 45°C there was a 50% decrease in growth rate (Fig 2B). In addition to the significant defects in vegetative growth, conidia form the *pkcA* ^G579R^ mutant showed a significant delay in germination. When germination was monitored in complete medium, there was a significant decrease (p ≤ 0.01) in the emergence of the germ-tubes in the *pkcA* ^G579R^ mutant of 50% at both 37°C and 45°C after 8 hours of growth (Fig 2C). The *pkcA* ^G579R^ mutant did not exhibit altered pigmentation or differences in the number of conidia formed either at 37°C or 45°C (data not shown). Taken together, these morphological analyses of the *pkcA* ^G579R^ mutant suggest that the impaired function of *pkcA* interferes in the hyphal growth and elongation.

### The *pkcA* gene is required for the maintenance of cell wall integrity, proper conidial architecture and oxidative stress tolerance

**Fig 3:**
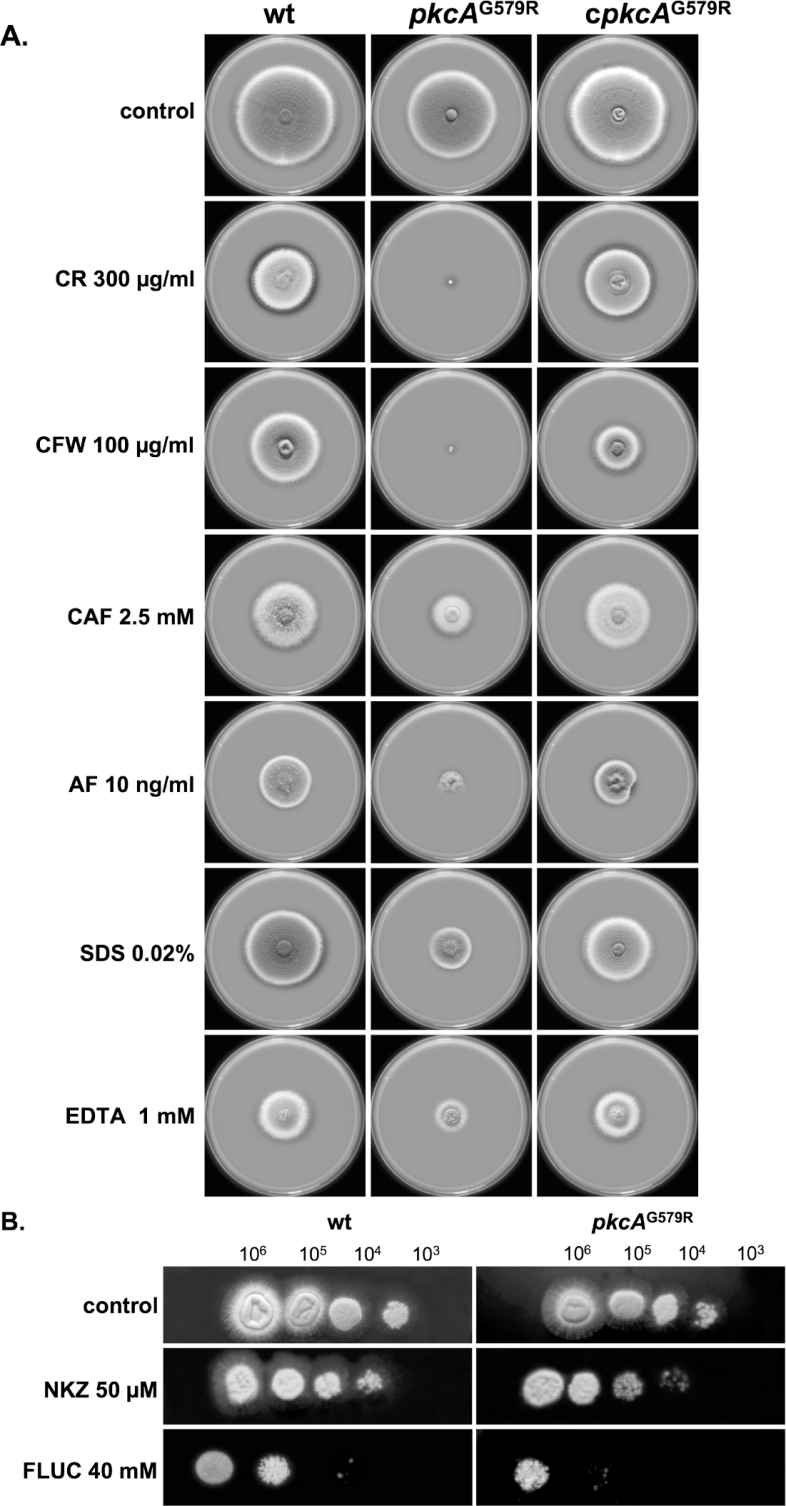
Growth phenotypes of the *pkcA* ^G579R^ mutant. (A) 1x10^5^ conidia of each strain were inoculated onto the center of a solid YG plates supplemented with different cell wall perturbing agents: Congo Red (CR); Calcofluor White (CFW); caffeine (CAF); anidulafungin (AF); Sodium Dodecyl Sulfate (SDS) and ethylenediaminetetraacetic acid (EDTA). (B) The indicated number of conidia was spotted onto solid YG plates supplemented with nikkomycin Z (NKZ) and fluconazole (FLUC). The plates were incubated for 3 days (A) or 2 days (B) at 37°C.

The involvement of the *pkcA* ^G579R^ mutation in cell wall biogenesis in *A*. *fumigatus* was further tested by utilizing several known cell wall perturbation agents. The *pkcA* ^G579R^ mutant showed increased sensitivity to the cell wall stressing agents Congo Red (CR), Calcofluor White (CFW) and the echinocandin anidulafungin (AF), plus to Sodium Dodecyl Sulfate (SDS), which disrupts the plasma membrane and lyses cells with membrane defects, and caffeine (CAF) that has been extensively used to probe signal transduction and cell integrity phenotypes in *S*. *cerevisiae* [62] (Fig 3A). Lower concentrations of CR and CFW were also tested (S2 Fig). A subtle increase in the sensitivity of the *pkcA* ^G579R^ mutant to the chitin synthase inhibitor, nikkomycin Z (NKZ), and to fluconazole (FLUC) was observed in comparison to the wild-type and complemented strains (Fig 3B). The *pkcA* ^G579R^ mutant also showed decreased tolerance to EDTA (Fig 3A).

**Fig 4:**
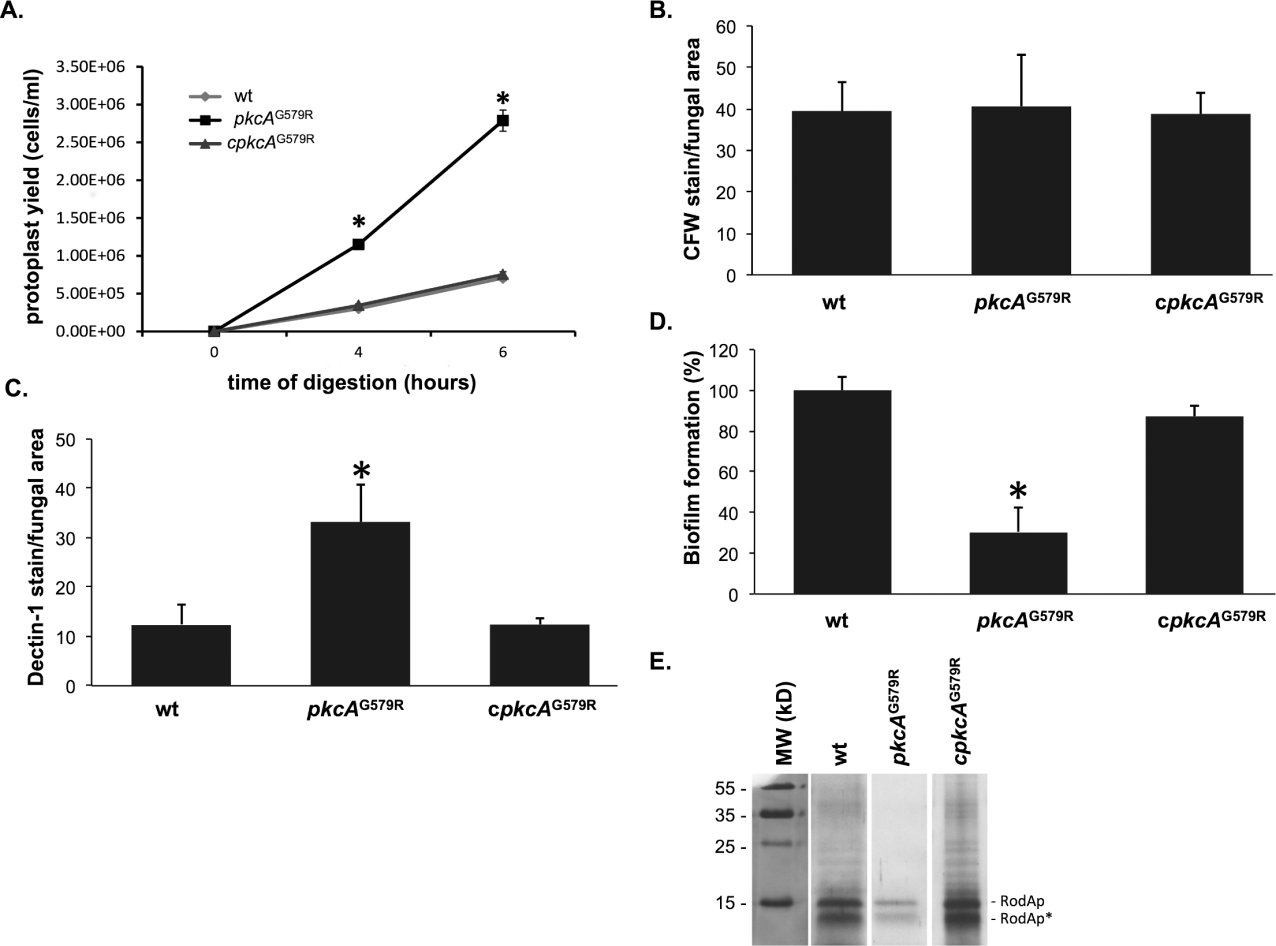
*pkcA* ^G579R^ mutant strain presents increased protoplasts production and β-1,3-glucan content, lower biofilm formation and reduced hydrophobin content. (A) The digestion mixture included the Lallzyme MMX and 100 mg of mycelium into 50 ml of osmotic stabilizer solution at the indicated incubation times. Protoplasts were quantified using a Neubauer chamber. Detection of the exposed chitin (B) and β-1,3−glucan (C) contents on the cell surface. Conidia were cultured in liquid MM to the hyphal stage, UV-fixed, and stained with CFW or soluble dectin-1to detect the content of exposed chitin and β-glucan, respectively. The intensity of staining was calculated by averaging the amount of staining to the total area of each fungal cell using ImageJ software. These experiments were performed in triplicate, and the results are displayed as mean values ± standard deviation. * Student’s T-test (p ≤ 0.05). (D) Biofilm formation was evaluated by crystal violet absorbance at 570 nm and expressed as the percentage of adhesion considering 100% for the wild-type strain. Experiments were performed in quintuplicate and the results are the mean ± standard deviation. (*p ≤ 0.01). (E) Silver stained SDS-PAGE profiles of HF acid extracts from resting conidia showing the hydrophobin concentration of the wild-type, *pkcA* ^G579R^ and complementing strains. The number of conidia subjected to the hydrophobin extraction was validated by CFU counting on solid YG medium. RodAp corresponds to the native RodA and RodAp* to partially degraded or processed RodA.

CR and CFW displayed the most pronounced effects on the *pkcA* ^G579R^ mutant. The increased sensitivity to CR and CFW phenotype could be partially rescued on minimal media containing the osmotic stabilizer sorbitol (1.2 M). In addition, this phenotypic rescue was complete in the presence of AF, but not evident in the presence of CAF (S3 Fig). We did not observe lysis or swelling phenotype of conidia during germination for the *pkcA* ^G579R^ mutant in the presence of any cell wall damaging compound (data not shown), although excessively swelling during germination was observed in *A*. *nidulans calc2* mutant [21]. As an indirect approach to investigate the cell wall composition and architecture of the *pkcA* ^G579R^ mutant strain, hyphae of the *pkcA* ^G579R^, wild-type and complemented strains, which were grown in liquid YG medium, were subjected to enzymatic digestion by Lallzyme MMX. The protoplasts were then harvested and counted. Digestion of the *pkcA* ^G579R^ mutant yielded about 4 times more protoplasts than the wild-type and complemented strains (Fig 4A) indicating that the *pkcA* ^G579R^ mutant cell wall was much more susceptible to the enzymatic degradation, suggesting that it possessed a modified carbohydrate composition or it is more enzymatically accessible. To investigate if this carbohydrate modification affected the cell wall organization, the fungal cell wall chitin and β-1,3-glucan levels were assessed in these three strains via CFW staining and soluble dectin-1 staining, respectively. The intensity of CFW staining per fungal area was similar in *pkcA* ^G579R^ mutant, wild-type and complementing strains (Fig 4B). In contrast, the *pkcA* ^G579R^ mutant has higher levels of β-1,3-glucans (about 2.7- fold) than the wild-type and complementing strains (Fig 4C). Collectively, these results suggest that the *pkcA* ^G579R^ mutant had altered cell wall organization. In addition to providing structural integrity to the fungal cell, the cell wall is the main structure forming interactions with host tissues. So, we subsequently investigated the ability of the *pkcA* ^G579R^ mutant to promote cell-to-cell adhesion measuring the initial stages of biofilm formation of mature hyphae in polystyrene plates. Biofilm formation was significantly reduced (60%) in the *pkcA* ^G579R^ mutant (Fig 4D) when compared to the wild-type and complemented strains. Collectively, these results suggest that the PkcA signaling cascade influences adherence to polystyrene and biofilm formation in *A*. *fumigatus* and also its cell wall composition. In order to investigate if this phenotype was correlated with lower amount of conidial hydrophobins, resting conidia of each strain were treated with hydrofluoric acid (HF) aiming to extract the hydrophobins. The SDS-PAGE analysis indicated that the *pkcA* ^G579R^ mutant had a lower amount of hydrophobins in comparison to the wild-type and complemented strains (Fig 4E). This alteration seems to be an event unrelated to the conidial developmental program in the *pkcA* ^G579R^ mutant, since no abnormalities in the conidiophore structures were observed when slide cultures were analyzed under lactophenol cotton blue staining (data not shown).

**Fig 5:**
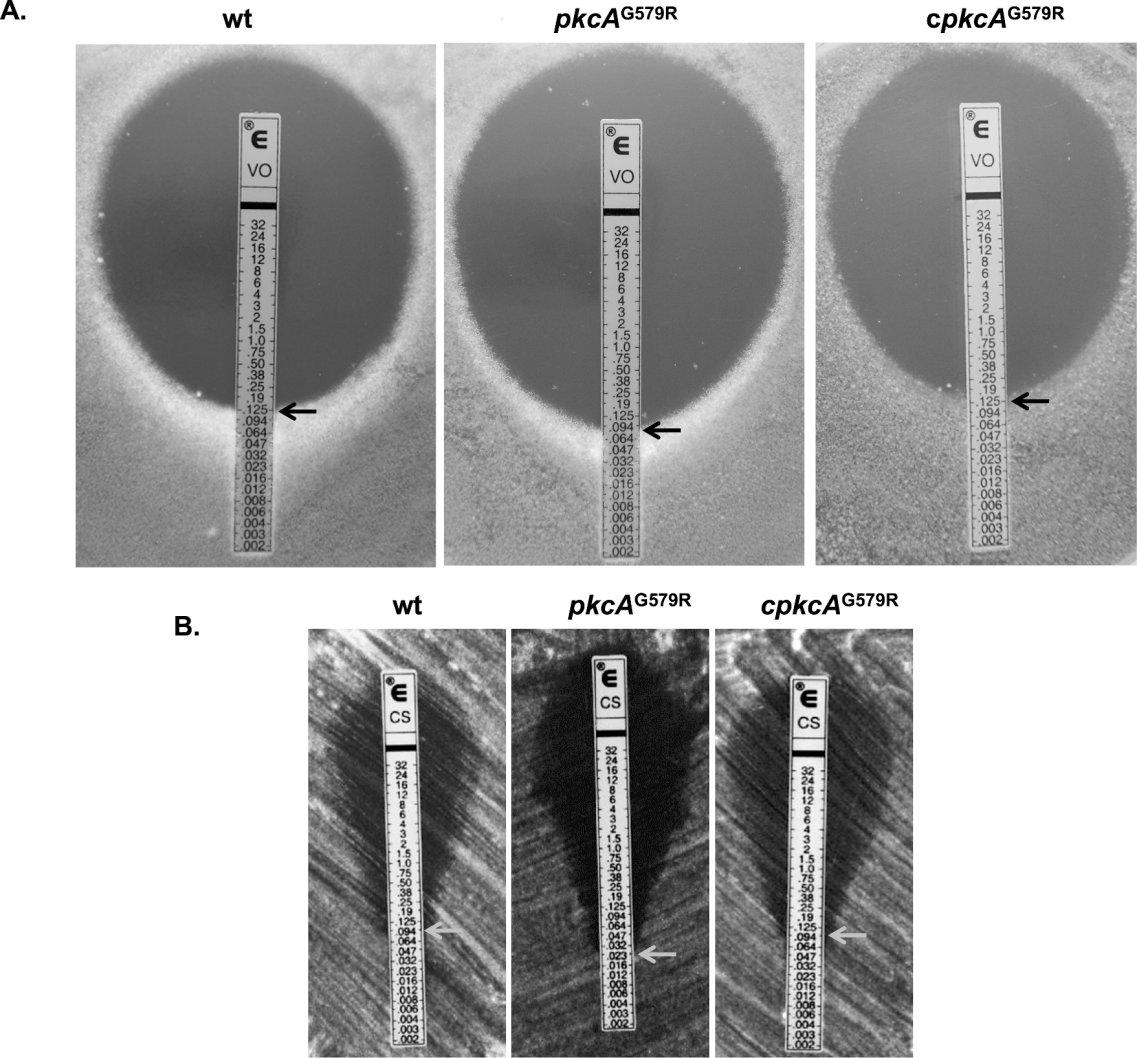
The *pkcA* ^G579R^ mutant is sensitive to antifungal drugs. Antifungal susceptibility using E-test gradient strips for voriconazole (A) and caspofungin (B).

Based on the cell wall-related phenotypes of the *pkcA* ^G579R^ mutant, we next examined the susceptibility of this strain, plus the wild-type and complemented strains, to the major classes of antifungal compounds in clinical use [63] via the E-test method, including voriconazole, amphotericin B and caspofungin (Fig 5). No differences were observed between the three strains for amphotericin B (data not shown) but there was a larger area of growth inhibition for the *pkcA* ^G579R^ mutant on voriconazole (endpoints 0.125 and 0.094 for wild-type and *pkcA* ^G579R^ mutant strain, respectively) and caspofungin (endpoints 0.094 and 0.023 for the wild-type *pkcA* ^G579R^ mutant strain, respectively) on the buffered RPMI medium. The caspofungin inhibition zone was clearer in the *pkcA* ^G579R^ mutant in contrast to the wild-type and complemented strains. Caspofungin is fungistatic to *A*. *fumigatus* forming a known incomplete clearing area around the caspofungin strip [43, 64, 65]. However, the clearer area obtained for the *pkcA* ^G579R^ mutant exposed to caspofungin may suggest that it can be fungicidal upon *pkcA* partial loss-of-function.

**Fig 6:**
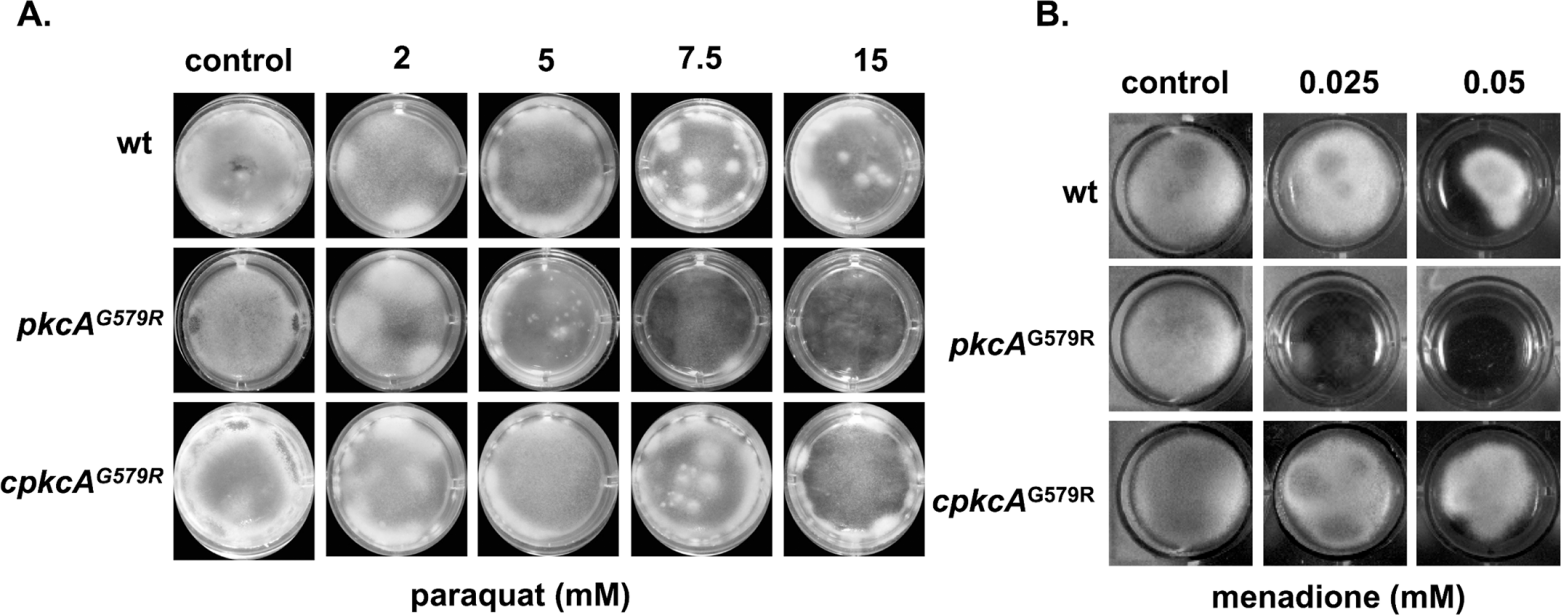
Growth phenotypes of the *pkcA* ^G579R^ mutant strain in the presence of oxidative damage. The strains were grown in liquid MM in 24-well plates supplemented with in the indicated concentrations of paraquat (A) or menadione (B) during 48 hours at 37°C.

Next, we investigate the response of the mutant to oxidative damage to get information if the *pkcA* could be indirectly involved in Reactive Oxygen Species (ROS) tolerance. The *pkcA* ^G579R^ mutant showed increased sensitivity to paraquat (PQT) and menadione in comparison to the wild-type and complemented strains (Fig 6). However, no effect was observed in the presence of H_2_O_2_ and diamide (data not shown). Taken together, these phenotypic analyses of the *pkcA* ^G579R^ mutant suggest that the *A*. *fumigatus pkcA* affects the ability of the fungus to form normal cell wall and its *bona fide* tolerance to oxidative damage elicited primarily by anion superoxide.

### The activation of the MAP kinase MpkA upon cell wall stress is defective in the *pkcA* ^G579R^ mutant

**Fig 7:**
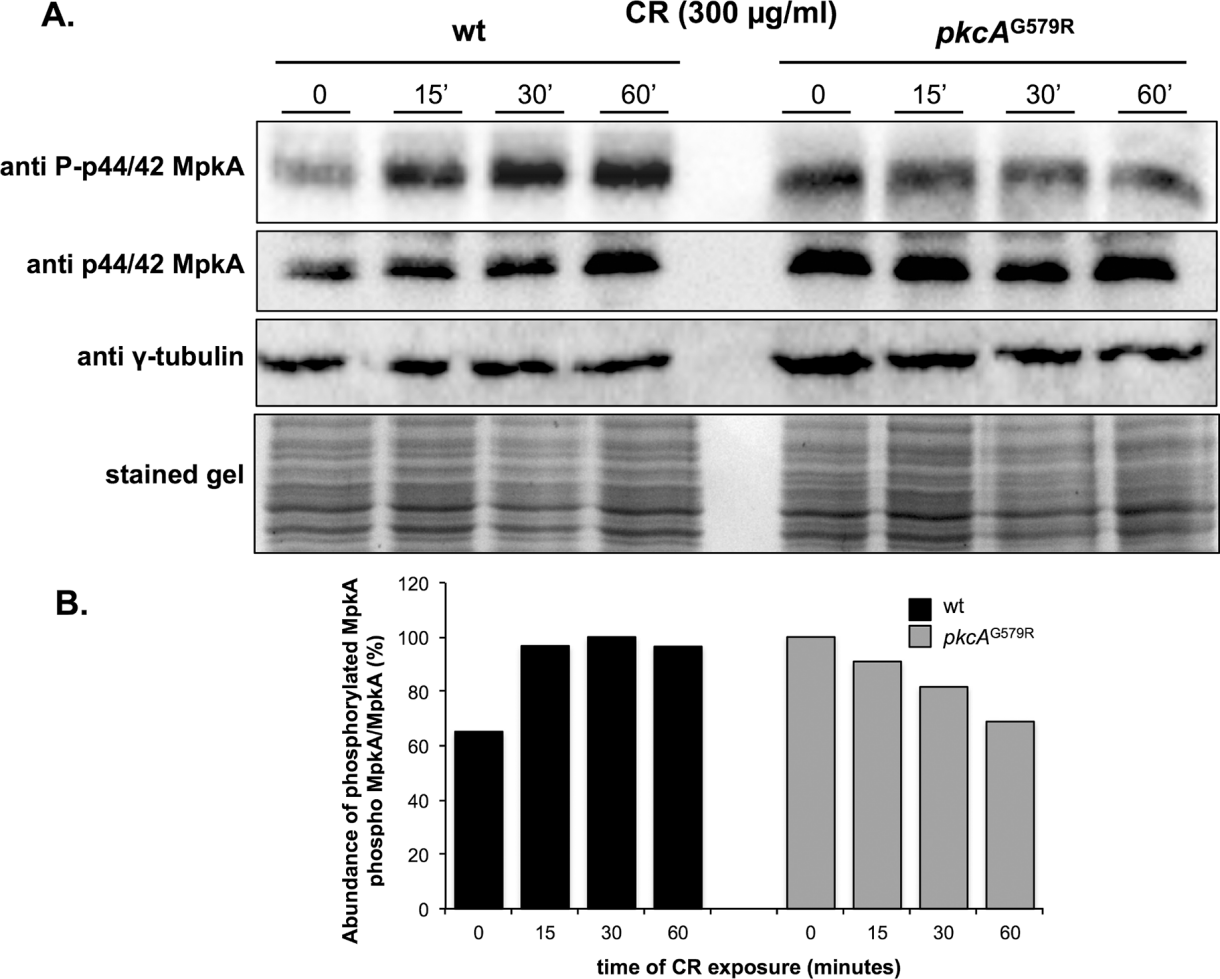
The *pkcA* ^G579R^ mutant strain is defective in activating the CWI pathway. Conidia of the wild-type and mutant strains were grown overnight in liquid YG medium and cell wall stress was induced by exposure to CR for 0, 15, 30 and 60 minutes. Samples were collected at the indicated time points for western blot preparation. Phosphorylated and the total fraction of MpkA were detected using anti-phospho p44/42 and anti-p44/42 MAPK antibodies, respectively. γ-tubulin antibody and Coomassie Brilliant Blue stained gel were used as loading sample controls (A). Densitometry analysis of western blots using the ImageJ software presenting the difference in the phosphorylated MpkA/non-phosphorylated MpkA ratio in the wild-type and mutant strain expressed as percentage in the histogram (B).

In *S*. *cerevisiae*, the PKC1 CWI pathway is activated by the exposure to cell wall stressing agents culminating with the Mpk1p (Slt2p) phosphorylation [66–68]. Although in *A*. *fumigatus* phosphorylation of the Mpk1p homologue, MpkA is achieved upon cell wall stress caused by Glucanex and CFW [16, 20] the requirement of PkcA in this signaling cascade was not investigated. Accordingly, the hypothesis that PkcA is acting upstream the MpkA for the activation of the CWI pathway was tested by the evaluation of the phosphorylation of MpkA upon CR-induced cell wall stress. CR was used to induce cell wall stress in the wild-type and *pkcA* ^G579R^ strains, for which the latter was highly sensitive to 300 μg/ml of this compound (Fig 3A and S2 Fig). The MpkA protein was phosphorylated in response to CR in the time course experiment presenting an increase of about 32%, 35% and 31% after 15, 30 and 60 minutes, respectively (Fig 7), indicating that CR can induce the CWI pathway in *A*. *fumigatus*. In contrast, the phosphorylation of MpkA was lower in the *pkcA* ^G579R^ mutant showing a decrease of about 10%, 19% and 31% after 15, 30 and 60 minutes post- CR treatment. Interestingly, the phosphorylation of MpkA was 47.3% higher in the *pkcA* ^G579R^ strain prior to the CR treatment. These results indicate that PkcA signals MpkA phosphorylation in the *A*. *fumigatus* CWI pathway.

### Loss-of-function of *pkcA* contributes to the Endoplasmic Reticulum (ER) stress condition during cell wall stress

**Fig 8:**
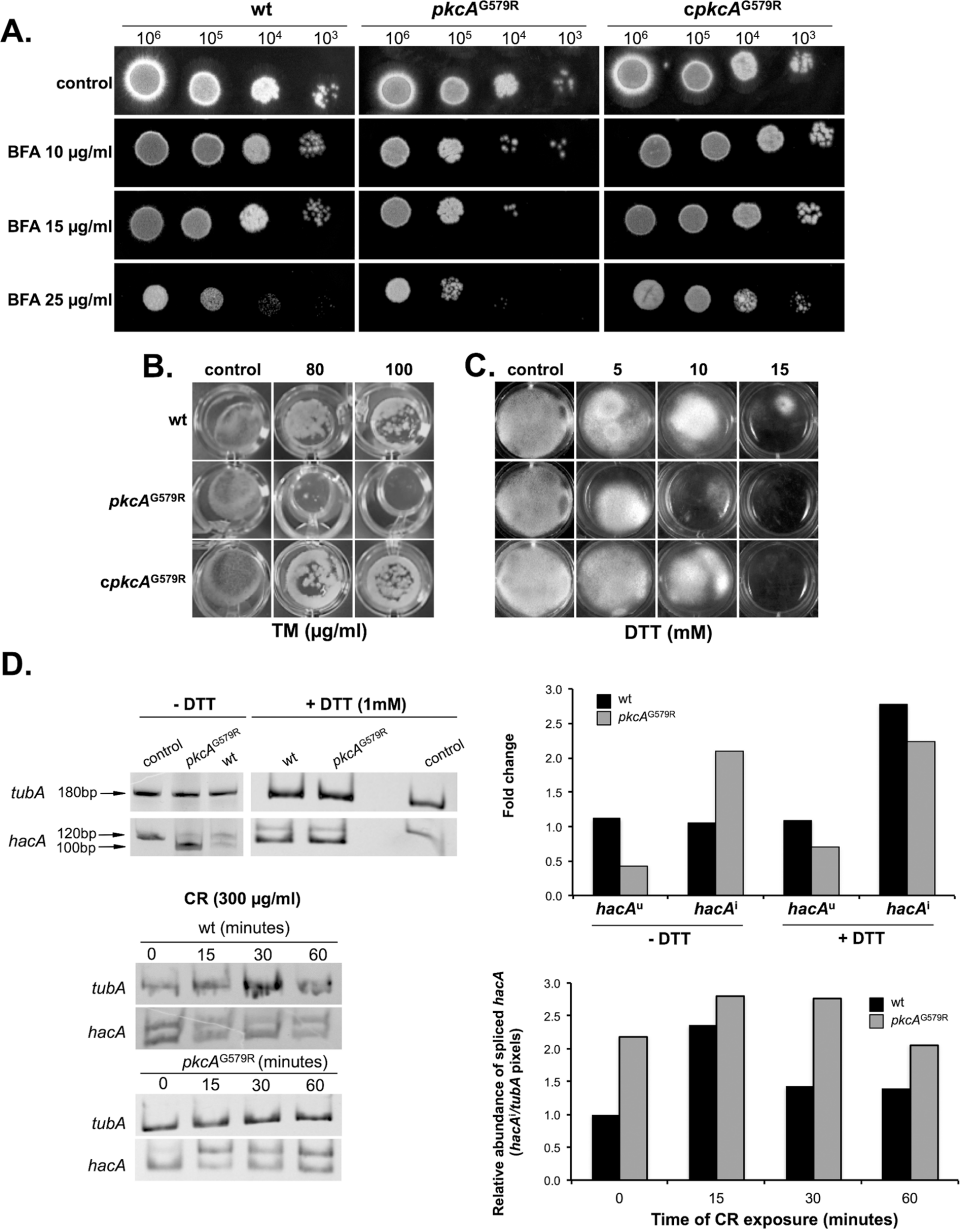
Cell wall stress caused by loss of *pkcA* is connected to UPR signaling in *A*. *FUMIGATUS*. (A) The indicated number of conidia of each strain was spotted on solid YG medium supplemented with BFA (brefeldin A) or in a 24 well plate (1x10^4^ conidia/well) containing the indicated concentration of TM (tunicamycin) (B); or (C) DTT. (D) Analysis of *hacA* mRNA splicing in the wild-type and *pkcA* ^G579R^ mutant. Overnight liquid cultures of each strain were subjected to cell wall stress by the addition of CR during the indicated amount of time before total RNA was extracted. RT-PCR was used to amplify the *hacA* transcript using primers that span de border of the 20 nucleotide unconventional intron to distinguish induced (spliced *hacA* form = *hacA* ^i^) from the uninduced (non-spliced *hacA* form = *hacA* ^u^) yielding fragments of 100 and 120 nucleotides, respectively. PCR products were separated in an acrylamide gel and stained. As a control, both wild-type and mutant strain were treated with 1 mM of DTT to induce UPR. Genomic DNA was also used as template in the same reactions yielding only the 120 bp band (upper panel). Images were subjected to densitometric analysis of pixel intensity and normalized by the expression of *tubA* run as loading control in each RT-PCR.

A connection between the cell wall integrity and the ER stress pathways has been described, since mutants defective in the activation of Unfolded Protein Response (UPR) are more sensitive to cell wall stressing agents [43, 46] and reviewed in [69, 70]. This relationship is supported by the fact that UPR maintains ER functionality via balancing the income of new proteins with processing capacity, which in turn influences secretion, cell wall homeostasis and ultimately fungal pathogenesis [43, 71, 72]. In *A*. *fumigatus* the canonical UPR is activated when misfolded proteins accumulate in the ER lumen leading to the oligomerization and activation of the cytosolic kinase and RNAse domains of the ER stress sensor IreA [46], which excises an unconventional intron (20 nucleotides) from the cytoplasmic mRNA of *hacA* (uninduced; *hacA* ^u^) [43]. The spliced mRNA of *hacA* (induced; *hacA* ^i^) can be translated in a fully functional bZIP transcription factor, which migrates to the nucleus allowing the transcription of genes related to the ER folding capacity [70]. Based on this information, we wanted to determine how the *pkcA* mutant is affected by ER-stressing agents such as DTT (dithiothreitol), tunicamycin (TM) and BFA (brefeldin A), which activate UPR by: unfolding proteins by reducing disulfide bonds, inhibiting N-linked glycosylation and interfering the anterograde protein transport form ER to the Golgi, respectively [73]. The *pkcA* ^G579R^ mutant was more sensitive to all ER stressing agents (Fig 8A–8C). In addition, the activation of *hacA* transcription factor was assessed by RT-PCR according to the assay previously described by [46]. Both wild-type and *pkcA* ^G579R^ strains were exposed to 1 mM of DTT during 60 minutes, which can activate UPR in *A*. *fumigatus* [43, 46]. Interestingly, in the *pkcA* ^G579R^ mutant strain, the UPR is activated under a condition without DTT as shown by the 2- fold increase in the *hacA* ^i^ transcript (Fig 8D, upper graph). To evaluate the mRNA abundance of *hacA* ^i^ during cell wall stress, the strains were exposed to CR. In the wild-type strain exposed to CR there was an increased accumulation of the *hacA* ^i^ mRNA (2.4- fold mRNA increase) after 15 minutes and a drop to 1.4- fold after 30 and 60 minutes. The *hacA* ^i^ mRNA accumulation in the *pkcA* ^G579R^ mutant was 2.2- fold higher than the wild-type strain prior to CR treatment (Fig 8D, lower graph). In addition, levels of *hacA* ^i^ were maintained at high levels (2.8-, 2.8- and 2.2- fold increase) post CR treatment (Fig 8D). These results suggest that cell wall defects elicited by the PkcA-mediated CWI pathway are directly connected to the ER stress and also provide experimental data reinforcing the hypothesis that the secretion system is fundamental for maintaining cell wall homeostasis. This idea is also supported by the fact that the increased sensitivity of the *pkcA* ^G579R^ mutant strain to DTT and BFA can be rescued to the wild-type levels when the medium is supplemented with D-sorbitol as osmotic stabilizer (S4 Fig).

### The *pkcA* ^G579R^ mutant shows an altered expression pattern of cell wall integrity-related genes upon exposure to cell wall stress

**Fig 9:**
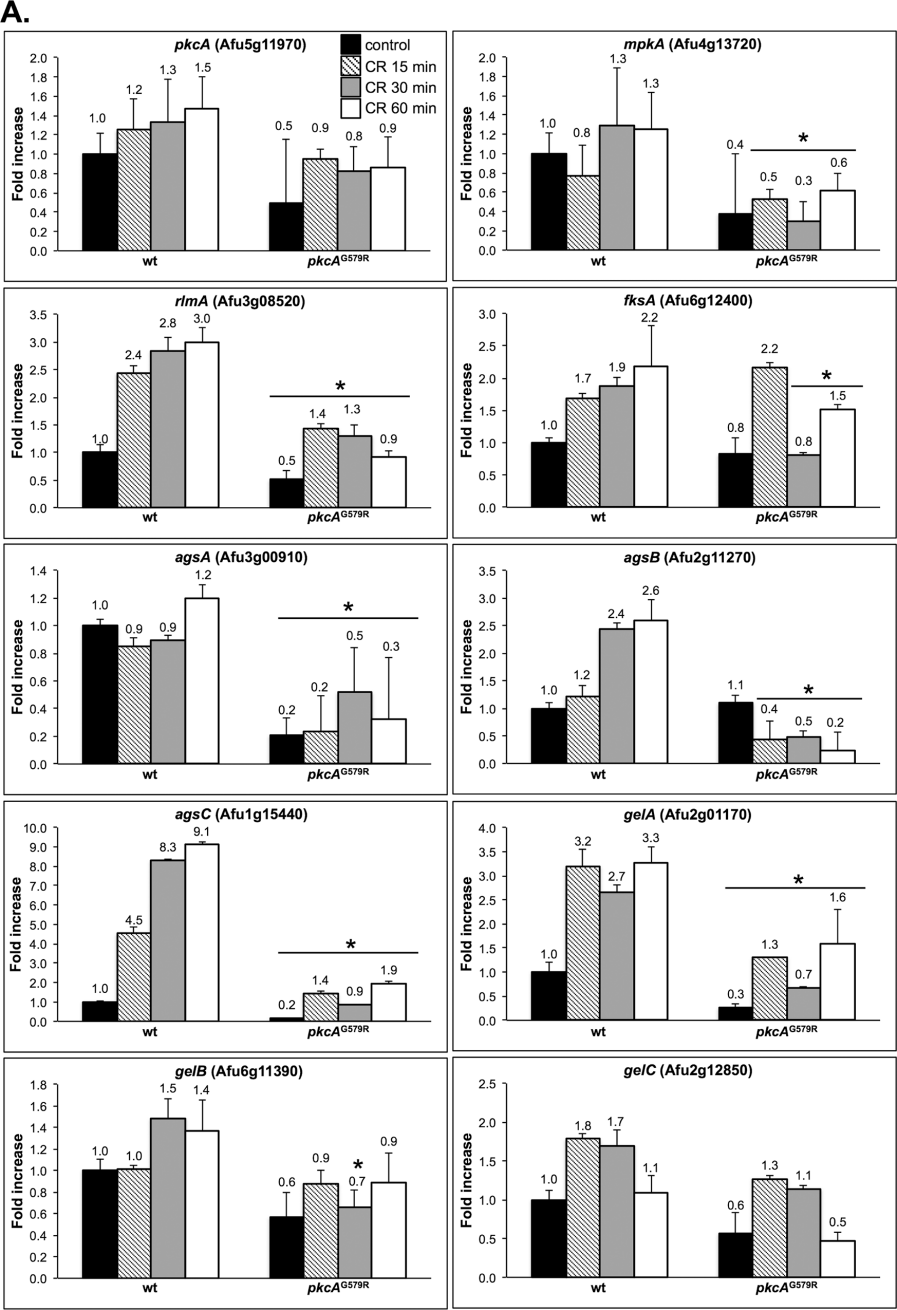
Transcriptional analysis of cell wall-related genes. The wild-type, and *pkcA* ^G579R^ strains were grown for 24 hours in YG medium and transferred to fresh pre-warmed YG with either 0 or 300 μg/ml of CR and grown for a further 15, 30 and 60 minutes. mRNA abundance for each gene was assessed by real time RT-PCR and normalized to β-tubulin. Fold increase in each strain represents the normalized mRNA abundance relative to the wild-type strain at time point 0 (i. e. prior to CR exposure). Data represent the average value of at least three biological replicates, each repeated in duplicate in the same run and standard deviation. * p ≤ 0.05.

**Fig 10:**
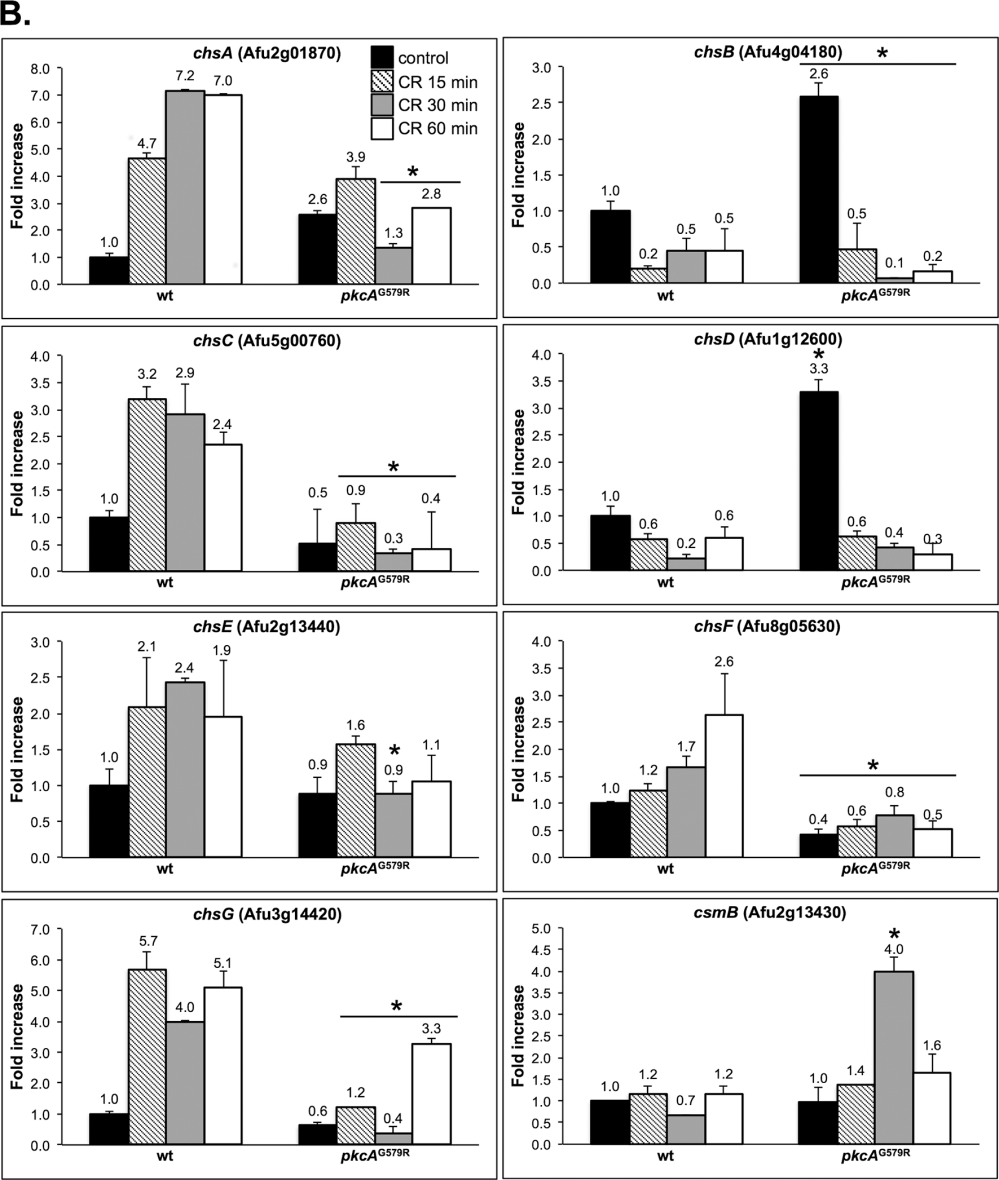
Transcriptional analysis of chitin synthase genes. The wild-type, and *pkcA* ^G579R^ strains were grown for 24 hours in YG medium and transferred to fresh pre-warmed YG with either 0 or 300 μg/ml of CR and grown for a further 15, 30 and 60 minutes. mRNA abundance for each gene was assessed by real time RT-PCR and normalized to β-tubulin. Fold increase in each strain represents the normalized mRNA abundance relative to the wild-type strain at time point 0 (i. e. prior to CR exposure). Data represent the average value of at least three biological replicates, each repeated in duplicate in the same run and standard deviation. * p ≤ 0.05.

The *A*. *fumigatus pkcA* ^G579R^ strain was shown to be more sensitive to several cell wall damaging compounds, and CR was able to induce a PkcA-dependent CWI pathway leading to the phosphorylation of MpkA (Fig 7). Hence, we used a transcriptional approach to investigate the cause of the increased sensitivity of the *pkcA* ^G579R^ mutant to cell wall stressing agents, via monitoring the expression of genes known to be involved in the cell wall biosynthesis and reinforcement in *A*. *fumigatus*. Hyphae from wild-type and *pkcA* ^G579R^ mutant strains were exposed to CR for 0, 15, 30 and 60 minutes prior to the quantification of mRNA abundance using real time RT-PCR. Accordingly, we examined the mRNA levels of *pkcA*, *mpkA*, and the Afu3g08520 (here named *rlmA*) that encodes the putative homologue of *S*. *cerevisiae RLM1* transcription factor [14, 18]. In addition, the expression of the main cell wall biosynthesis genes: α-1,3-glucan synthases (*agsA-C*); β-1,3-glucan synthase (*fksA*); 1,3-β-glucanosyl transferases (*gelA-C*), and the eight *A*. *fumigatus* chitin synthases (*chsA-G* and *csmB*), were also investigated (Figs 9 and 10).

Upon CR treatment, *pkcA* was slightly induced, while *mpkA* transcript levels were increased in the wild-type strain after 30 and 60 minutes CR treatment. Interestingly, there was lower abundance of *mpkA* transcripts in the *pkcA* ^G579R^ mutant compared to the wild-type strain (0.3- and 1.3- fold increase after 30 minutes, respectively). This correlated with the lower level of MpkA phosphorylation under the same growth conditions (Fig 7). Likewise, levels of the *rlmA* transcription factor were up-regulated in the wild-type strain in a time-dependent manner, while *rlmA* expression was significantly lower in the *pkcA* ^G579R^ mutant in the presence or absence of CR (Fig 9), suggesting that the activation of *rlmA* in response to CR depends on the PkcA-MpkA pathway.

Regarding the cell wall biosynthetic enzymes assessed in this study, all the genes showed increased expression in the wild-type strain, at different levels for at least one time point, except for the chitin synthases *chsB*, *chsD* that were repressed and *csmB* that showed similar expression during CR exposure. Interestingly *chsB* and *chsD* were expressed at a higher level in the *pkcA* ^G579R^ mutant in the absence of CR (2.6- and 3.3- fold increase, respectively), indicating that these two genes may be critical for the cell to cope with cell wall stress imposed solely by the loss-of-function to *pkcA* (Fig 10). Levels of the catalytic subunit of the β-1,3-glucan synthase *fksA* were also increased in a time dependent-manner in the wild-type strain reaching 2.2- fold induction after 60 minutes of CR exposure. In contrast different values were observed in the *pkcA* ^G579R^ mutant especially after 30 and 60 minutes of treatment (0.8- and 1.5- fold induction). This was expected due to the increased sensitivity of the *pkcA* ^G579R^ mutant to caspofungin and anidulafungin, which indicated altered β-1,3-glucan and β-1,6-glucan synthesis. However, the induction of *fksA* in the *pkcA* ^G579R^ mutant after 15 minutes of CR treatment (2.1- fold increase) indicates that it was not completely affected by the *pkcA* mutation, suggesting that either residual activity of PkcA^G579R^ can stimulate its activation, or *fksA* expression does not depend on PkcA-MpkA signaling cascade.

Relevant differences in the transcript levels were observed in the three α-1,3-glucan synthase encoding genes, *agsA*, *agsB* and *agsC*. Interestingly, *agsA* mRNA was maintained at low levels regardless of CR exposure, but the transcription of *agsB* and *agsC* were increased after CR stress in the wild-type strain. The largest fold change was observed for *agsC*, which was increased over 4.5-, 8.2- and 9.1- fold after 15, 30 and 60 minutes of CR treatment respectively. On the other hand, the transcription of *agsB* and *agsC* in the *pkcA* ^G579R^ mutant was markedly reduced to 0.2- and 1.9- fold, respectively after 60 minutes of incubation (Fig 9). These results suggest that the transcription of α-1,3-glucan synthase depends on the PkcA-MpkA signaling transduction in *A*. *fumigatus*.

The mRNA levels of the genes encoding cell wall remodeling enzymes, *gelA*, *gelB* and *gelC*, were also analyzed. No significant differences were observed between the wild-type and *pkcA* ^G579R^ mutant strains for the *gelB* and *gelC*. In contrast, the *gelA* transcript levels in the wild-type strain, but not the *pkcA* ^G579R^ mutant, increased in all time points post CR treatment. This also indicates that the transcription of *gelA* depends on PkcA kinase activity. Chitin synthase encoding genes, *chsA* and *chsG*, showed higher induction after CR exposure (6.9- and 5.1- fold, respectively at 60 minutes) and this induction was clearly lower in the *pkcA* ^G579R^ mutant at the same time point (2.8- and 3.2- fold, respectively). The same was observed for *chsC* and c*hsF* in all time points (Fig 10). Interestingly, *csmB* showed similar expression profile in the *pkcA* ^G579R^ mutant in comparison to the wild-type strain, except for the timepoint 30 minutes where there was a 3.9- fold induction. Taken together, these data suggest that PkcA contributes to the transcriptional regulation of several but not all cell wall-related genes in *A*. *fumigatus*.

### The *pkcA* ^G579R^ mutant elicits increased TNF−α levels and macrophage recognition but it is not able to disturb virulence in a low dose neutropenic murine infection model

**Fig 11:**
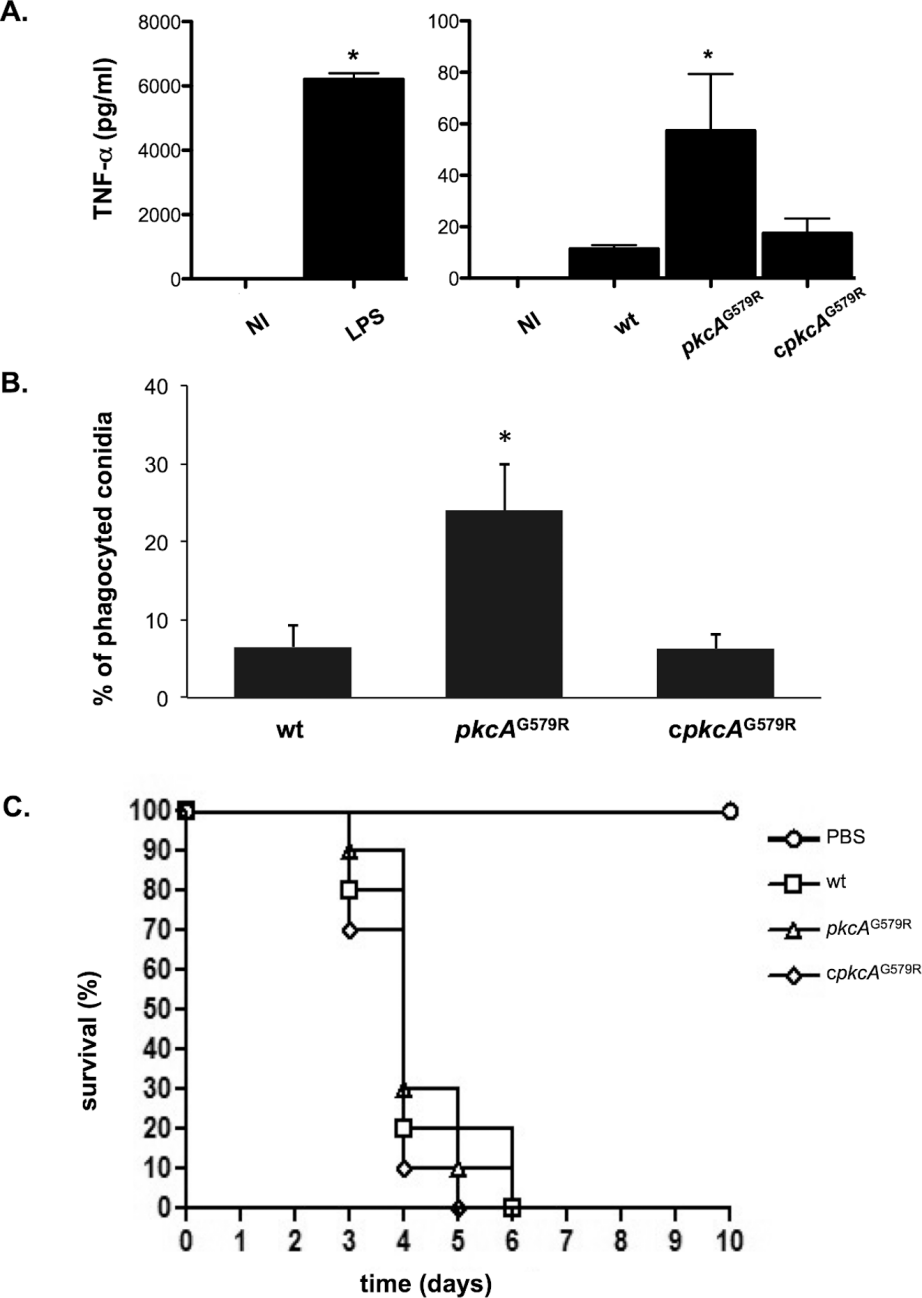
*pkcA* ^G579R^ strain presents no virulence attenuation in a mouse model but activates innate immunity against *A*. *fumigatus*. (A) Secretion of TNF-α from bone marrow derived macrophages (BMDM) after co-incubation with *A*. *fumigatus* hyphae of wild-type, *pkcA* ^G579R^ and complementing strains. TNF-α levels were quantified by ELISA from the culture supernatant after 18 hours of BMDMs infection. Data show average ± standard deviation (* p ≤ 0.005). NI: Non−infected; LPS: lipopolysaccharide (positive control). (B) Phagocytosis index is increased in the *pkcA* ^G579R^ mutant strain (average ± standard deviation, *p ≤ 0.01 compared to the wild−type and the complemented strain). (C) Comparative analysis of wild type, *pkcA* ^G579R^ and complemented strains in a neutropenic murine model of invasive pulmonary aspergillosis.

The *pkcA* ^G579R^ mutant has an impaired response to cell wall stress and alterations to the conidia surface. Thus, we reasoned if these phenotypes would impact on the host immune response. So, we used Bone Marrow Derived Macrophages (BMDMs) to measure the levels of the proinflammatory cytokine Tumor Necrosis Factor alpha (TNF−α) released by these cells after co-incubation with *A*. *fumigatus* conidia. TNF−α is an important inflammatory mediator secreted by macrophages when exposed to *A*. *fumigatus* [74, 75]. BMDMs co-cultured with *pkcA* ^G579R^ strain showed about 4- fold higher TNF−α production than the wild−type or the complemented strain (Fig 11A). We also test the ability of BMDM in internalizing wild-type, mutant and completing strain conidia. About 25% of the *pkcA* ^G579R^ strain conidia were phagocytized after the 80 minutes of co-incubation. In contrast, only 6.3% of the wild-type and complementing strains conidia were internalized (Fig 11B). These results suggest that the effect caused by *pkcA* ^G579R^ mutation on the *A*. *fumigatus* CWI is important for macrophage recognition and inflammatory responses. To determine the possible influence of *pkcA* on virulence, the wild-type, *pkcA* ^G579R^ mutant and the complemented strains were compared in a murine model for invasive aspergillosis [56]. In spite of the higher TNF-α elicited production and the increased phagocytosis levels in the *pkcA* ^G579R^ mutant, all the strains caused the same absolute mortality and similar survival kinetics after the immunosuppressive regimen and intranasal infection after five to six days (Fig 11C). These results indicate that although PkcA^G579R^ influences macrophage recognition, it does not affect fungal survival and virulence in the immunocompromised mammalian host.

## Discussion

The fungal CWI pathway that orchestrates cell wall biosynthesis and remodeling is evolutionary conserved in many fungal organisms. In *A*. *fumigatus*, this pathway is partially characterized, but how it is activated is not completely understood yet. Here, we characterized the *pkcA* gene of *A*. *fumigatus*, which encodes the protein kinase C, the apical kinase of the CWI pathway. Previous studies assessing the function of PKC in other human pathogenic fungi have revealed the importance of this gene in fungal pathogenesis, affecting the biosynthesis of virulence determinants such as melanin and a capsule in *C*. *neoformans* [28, 76, 77] and the tolerance to azoles in *Candida albicans* [78]. PKC is not essential in these two fungal pathogens although in *C*. *neoformans* Δ*PKC1* is only viable in the presence of D-sorbitol [77, 79]. In contrast, our results suggest that *pkcA* is essential in *A*. *fumigatus*, as was the case in *A*. *nidulans*. The *calC2* mutation described in the *A*. *nidulans pkcA* indicated that this gene is involved in the maintenance of the cell wall since it showed phenotypes related to cell wall defects [21]. The *pkcA* ^G579R^ mutant in *A*. *fumigatus* carries the same mutation as the *A*. *nidulans calC2* strain. This mutation is located beside the C-terminal limit of the cysteine-rich C1B regulatory domain, very close to the PkcA catalytic site, which can explain the cell wall defects and the fungicidial effect of the PKC inhibitor, chelerythrine associated with this mutation.

Here, the *pkcA* ^G579R^ mutation resulted in restricted vegetative growth. In *A*. *fumigatus*, the downstream components of the CWI, (*mpkA*, *bck1* and *mkk2*) and some upstream mechanosensors such as *midA* also play a role in the hyphal elongation [16, 20]. Collectively, these observations indicate that impairment of the CWI pathway impacts not only the cell wall composition, but also filamentous growth. Interestingly, asexual sporulation was not affected in the mutant strain, although we have observed that surface rodlets and hydrophobins were significantly reduced in the *pkcA* ^G579R^ mutant. It was demonstrated that the α-glucan synthase triple mutant (Δ*agsA-C*) also presented an amorphous surface without the rodlet layer [80]. Here, we observed lower levels of *agsA-C* expression upon CR exposure for the *pkcA* ^G579R^ mutant in comparison to the wild-type strain (Fig 9) indicating that PkcA might govern conidia surface structure organization, possibly representing an indirect effect of the perturbed CWI pathway resulting from the PkcA mutation. Recently we have shown that the *A*. *fumigatus* phosphatase *sitA*, modulates the activity of PkcA. The Δ*sitA* mutant shows decreased protein kinase C activity, increased sensitivity to cell wall damaging agents and also reduced adhesion properties and lower hydrophobin content [81]. The results presented here strongly suggest an epistatic relationship between *sitA* and *pkcA* and support the idea that SitA is contributing to the regulation of *A*. *fumigatus* CWI pathway. We are currently investigating this hypothesis.

Further evidence for a major cell wall defect in the *pkcA* ^G579R^ mutant was shown by the increased sensitivity of this strain to several cell wall perturbing agents, in particular the β-1,3-glucan synthesis inhibitors and agents that either target chitin biosynthesis (NKZ) or the linkage of chitin polymers to β-1,3-glucan and β-1,6-glucan (CFW and CR) [82, 83]. Together, these results suggest that the compromised function of *pkcA* results in a significant alteration in both chitin and β-glucan biosynthesis, or the ability to properly assemble the cell wall matrix. This conclusion is also supported by (i) the increased production of protoplasts upon digestion of *pkcA* ^G579R^ hyphae, (ii) the partial growth recovery on D-sorbitol, and (iii) the increased β-1,3-glucan levels in the mutant strain. These results emphasize that changes in β-glucan and chitin architecture or chitin/β-glucan cross-links, are possibly responsible for the observed phenotypes. However, quantitative analyses of cell wall components in *pkcA* ^G579R^ mutant stay for future investigation.

*A*. *fumigatus pkcA* coordinates responses to different cell wall stresses and also to oxidative damage. Our results showed the increased sensitivity of the *pkcA* ^G579R^ mutant to oxidative stressing agents, paraquat and menadione, but not to H_2_O_2_. *C*. *neoformans* PKC1 is essential for the protection against both oxidative and nitrosative stresses caused by H_2_O_2_, diamide or NaNO_3_, respectively. In *C*. *neoformans* the oxidative stress signal results in PKC1 and subsequently Mpk1 activation. However, the other downstream PKC1 elements in the *C*. *neoformans* CWI pathway are dispensable for resistance to oxidative and nitrosative stresses [77]. Considering the mode of action of menadione and paraquat are different from H_2_O_2_ [84–86], *pkcA* seems to be involved in ROS detoxification caused by superoxide anion (•O_2_^-^) which is involved in mitochondrial function, instead of the hydroxyl radicals (•OH) generated by H_2_O_2_ via Fenton reaction [87].

The UPR has been demonstrated to be involved in virulence in several human pathogenic fungi, including *A*. *fumigatus*. UPR influences important cellular functions such as morphology, thermo-tolerance, hypoxia, iron acquisition and tolerance to cell wall stress [43, 46, 71, 72, 88]. Here we demonstrated that impairment of the *A*. *fumigatus* PkcA-MpkA signaling pathway resulted in the activation of the UPR, indicating that both systems occur concomitantly and are reciprocally affected [69]. This connection between the CWI pathway and the UPR has previously been observed in *S*. *cerevisiae*, but only for different components of the CWI pathway, including the mechanosensor Mid1 and Mpk1 [72]. In *A*. *nidulans* the involvement protein kinase C in farnesol tolerance is related to the UPR [34], while elevated MpkA phosphorylation also correlates with increased tolerance to ER stress/ UPR inducing compounds [89]. Therefore, our results suggest that this connection is conserved in fungi and reinforces the role of PkcA in the *A*. *fumigatus* CWI pathway.

There is a great deal of speculation about which genes involved in the synthesis and remodeling of the cell wall are under the control of the canonical CWI pathway in *Aspergillus* species. Here, we aimed to identify some genes that are controlled by the *A*. *fumigatus* PkcA-MpkA pathway since the coordinated expression of these genetic determinants ultimately contributes to the virulence of this pathogen. It has been pointed out differences between *S*. *cerevisiae* and *A*. *fumigatus* [18, 69]. In *S*. *cerevisiae*, the canonical CWI pathway is responsible for the control of the vast majority of cell wall related genes [90], including proteins, which are not present in *A*. *fumigatus*, such as the Pir family proteins. Likewise, the cell wall polysaccharide, α-1,3-glucan, is not produced by *S*. *cerevisiae* or *C*. *albicans*, but is the most abundant polysaccharide in the cell wall of *A*. *fumigatus* [7]. So, it is reasonable that these differences in carbohydrate composition between these fungi, may explain the variations in the signaling processes. Here, the clearest example of PkcA-dependent gene expression was observed for the *agsA-C* genes, as expression was dramatically reduced in the *pkcA* ^G579R^ mutant. A detailed study by Fujioka *et al*. (2007) in the *A*. *nidulans* wild-type, Δ*mpkA* and Δ*rlmA* strains, challenged with micafungin, revealed that *agsB* (the homologue of *A*. *fumigatus agsA*) was also down-regulated in both mutant backgrounds. In contrast to our results, in *A*. *nidulans*, *agsA* (the homologue of *A*. *fumigatus agsB*) was up-regulated in both mutants. *A*. *nidulans* does not have an ortholog of *A*. *fumigatus agsC*, which was the most highly expressed α-glucan synthase encoding gene post exposure to CR in this study. *A*. *niger agsA* is the closest homolog of *A*. *fumigatus agsC*. In *A*. *niger*, *agsA* also showed the highest level of expression, among the five α-1,3-glucan synthase encoding genes, post exposure to CFW [91]. Interestingly, only the deletion of *agsC* can cause virulence attenuation in *A*. *fumigatus* [92, 93]. As expected, the regulation of seven chitin synthase encoding genes was also altered in the *pkcA* ^G579R^ mutant. However only *chsC*, *chsF* and *chsG* showed significantly lower transcription after CR treatment in the mutant strain. These results suggest that regulation of chitin synthase encoding genes occurs either in a CWI-dependent and-independent manner.

Echinocandins are the only class of drugs currently targeting an enzyme involved in cell wall biosynthesis, i.e. *fksA*. The *fksA* gene was induced upon cell wall stress caused by micafungin or CFW in both *A*. *nidulans* and *A*. *niger*, respectively [18, 94]. Recent data indicated that *fksA* is not an essential gene in *A*. *fumigatus*, which may partly explain the limited antifungal activity of the echinocandins against *A*. *fumigatus* [95]. Here, changes in *fksA* mRNA levels after CR treatment were similar in the wild-type and *pkcA* ^G579R^ strains. These results suggest that the basal levels of transcription of this gene and the increased expression after CR exposure occurs independently of the *A*. *fumigatus* PkcA. Altogether, the transcriptional profiling shown here indicates that a minor transcriptional response can be attributable to the canonical CWI pathway via *A*. *fumigatus* PkcA-MpkA cascade. This suggests that one or more signaling pathways might govern the expression of some CWI genes in response to CR. Therefore, functionally distinct signaling pathways induce a more efficient response to cope with a specific cell wall stress and damage. This conclusion can also support the observations that the fungus can alter its cell wall content in response cell wall stress that perturb either glucan or chitin synthesis [96–98].

We have shown that *pkcA* ^G579R^ mutant has normal virulence in a murine model of pulmonary aspergillosis and is increasingly recognized by macrophages. TNF−α, one of the key inflammatory mediators secreted by macrophages in response to fungal hyphae, was increased in *pkcA* ^G579R^ mutant compared to wild-type and complemented strain. This pro−inflamatory cytokine plays an important role in the induction of the innate immune response to *A*. *fumigatus* [74, 75]. The possible modifications in the cell wall carbohydrates and proteins, reduced hydrophobin content, and other modifications in the *pkcA* ^G579R^ mutant cell wall could contribute to an increased recognition of the fungus by dectin-1 receptor. This could favor its increased phagocytosis by alveolar macrophages, and consequently the increased TNF-α production, since the binding of the receptor dectin-1 to fibrillar **β**-1,3 glucan is a major host fungal interaction during *in vitro* and *in vivo* infection [99–102]. Among the fungal pathogens where PKC homologs have been characterized via gene deletion/mutation studies, only in *C*. *albicans* did the PKC mutant show virulence attenuation [78]. Despite the cell wall related phenotypes presented by the *A*. *fumigatus pkcA* ^G579R^ mutant, no difference in the virulence was observed in comparison to the wild-type and complemented strains. We cannot rule out the possibility that this can be the result of the partial loss-of-function caused by the point mutation in the C1B domain of the polypeptide. However, MpkA, which is part of the regulatory circuit of PkcA, was also dispensable for virulence, although the cell wall related phenotypes of the Δ*mpkA* strain were even more severe than *pkcA* ^G579R^ mutation [19]. These observations strongly indicate that redundant mechanism may account for the CWI in *A*. *fumigatus*. This idea can also be supported by the increased immune response observed in macrophages exposed to *pkcA* ^G579R^ strain conidia, which is not directly translated into virulence attenuation.

In summary, functional analysis of PkcA in *A*. *fumigatus* indicated its role in the CWI pathway and in the tolerance to cell wall and oxidative damage. This suggests PkcA functions as a key regulator with multiple roles in the cell. Our results confirm previous suggestions that CWI genes still hold great potential for antifungal drug development especially considering the fact that the only drug acting directly on the cell wall homeostasis is not controlled by PkcA signaling cascade in *A*. *fumigatus*.

## Supporting Information

S1 Fig Generation of the *pkcA* ^G579R^ mutant and reconstituted strain. The *pkcA* genomic sequence was replaced by a cassette containing the full-length sequence of *pkcA* open reading frame and the G2044C (G579R) mutation. The *pyrG* auxotrophic marker was inserted 250 bp downstream of the *pkcA* stop codon. The cassette was constructed by *in vivo* recombination in *S*. *cerevisiae* (A). Southern blot analysis of *Xho*I-digested genomic DNA using probe which binds specifically to the *pkcA* 5’-region as indicated in (A) identified the predicted 9.2 and 5.1 Kb band in the wild-type and *pkcA* ^G579R^ mutant, respectively (B). Successful complementation of *pkcA* gene was confirmed in CR-resistant monoconidial transformants by PCR using primers pkcA GC FW and Afu5g11970 3R (C).
(PDF)

S2 Fig Growth phenotypes of the *pkcA* ^G579R^ mutant strain in the presence of CR and CFW. The indicates number of conidia in a 5 μl volume were inoculated in solid YG medium supplemented with CR and CFW. Plates were incubated at 37°C for 3 days.
(PDF)

S3 Fig Growth phenotypes of the *pkcA* ^G579R^ mutant in the presence of the osmotic stabilizer D-sorbitol. The indicated number of conidia was spotted onto solid MM at 37°C or 45°C, with or without 1.2 M of sorbitol, supplemented with CAF, CR, CFW and AF. The plates were incubated for 2 days at 37°C.
(PDF)

S4 Fig The *pkcA* ^G579R^ strain sensitivity to ER-stressing agents DTT and brefeldin A can be rescued by D-sorbitol. The indicated number of conidia was spotted onto solid MM, with or without 1.2 M of sorbitol, supplemented with brefeldin A (BFA). (B) 1x10^4^ conidia were inoculated in 1 ml of liquid MM in a 24 well plate supplemented with DTT, with or without 1.2 M of sorbitol. The plates were incubated for 3 days at 37°C.
(PDF)

S1 Table Primers used in this study for mutant construction. (PDF)

S2 Table Real-time PCR primers used in this study. (PDF)
