# Supplementary figures and images for: The Aspergillus fumigatus pkcA G579R Mutant Is Defective in the Activation of the Cell Wall Integrity Pathway but Is Dispensable for Virulence in a Neutropenic Mouse Infection Model
Source: PLoS One. 2015 Aug 21;10(8):e0135195. doi: 10.1371/journal.pone.0135195 (PMC4546635; doi:10.1371/journal.pone.0135195)

A.

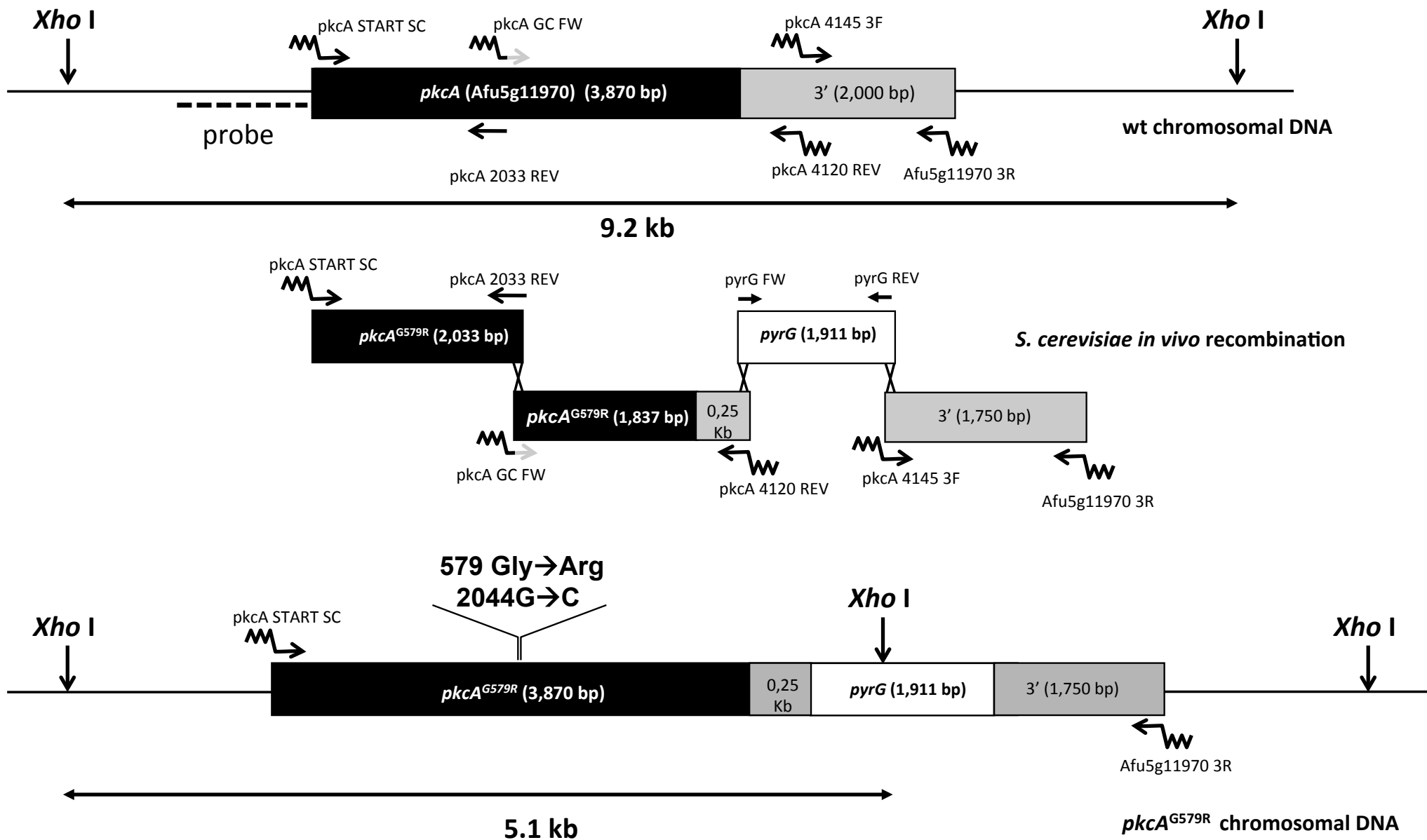

B.

wt

*pkcA*<sup>G579R</sup>

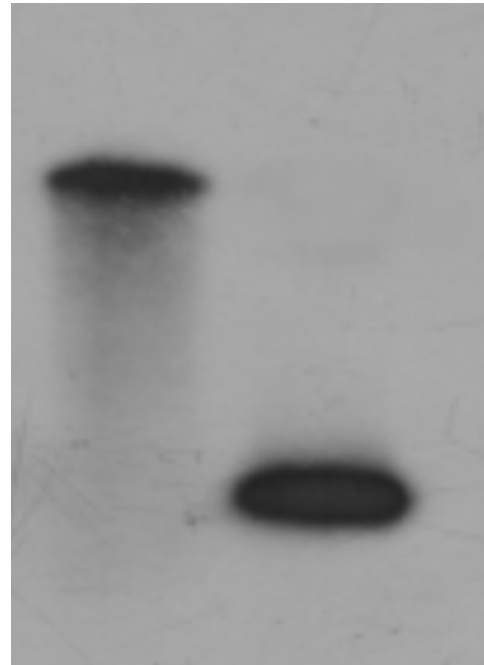

C.

wt

*pkcA*<sup>G579R</sup>

*cpkcA*<sup>G579R</sup>

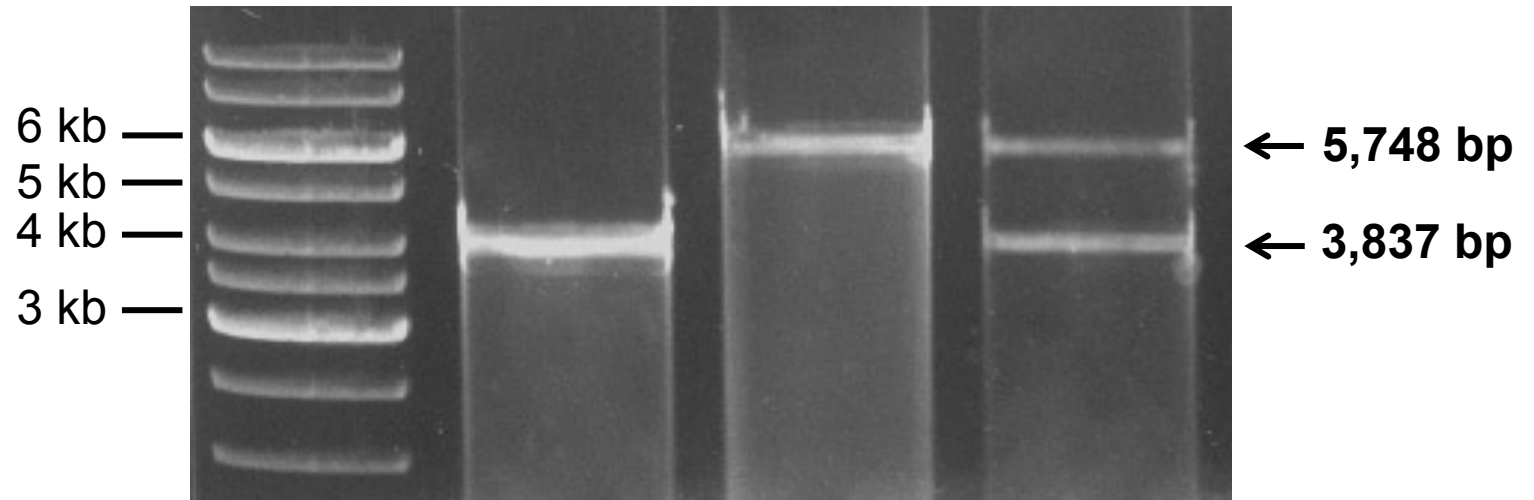

Supplement: S1 Fig — The pkcA genomic sequence was replaced by a cassette containing the full-length sequence of pkcA open reading frame and the G2044C (G579R) mutation. The pyrG auxotrophic marker was inserted 250 bp downstream of the pkcA stop codon. The cassette was constructed by in vivo recombination in S. cerevisiae (A). Southern blot analysis of XhoI-digested genomic DNA using probe which binds specifically to the pkcA 5’-region as indicated in (A) identified the predicted 9.2 and 5.1 Kb band in the wild-type and pkcA G579R mutant, respectively (B). Successful complementation of pkcA gene was confirmed in CR-resistant monoconidial transformants by PCR using primers pkcA GC FW and Afu5g11970 3R (C). (PDF) [file pone.0135195.s001.pdf]

A.

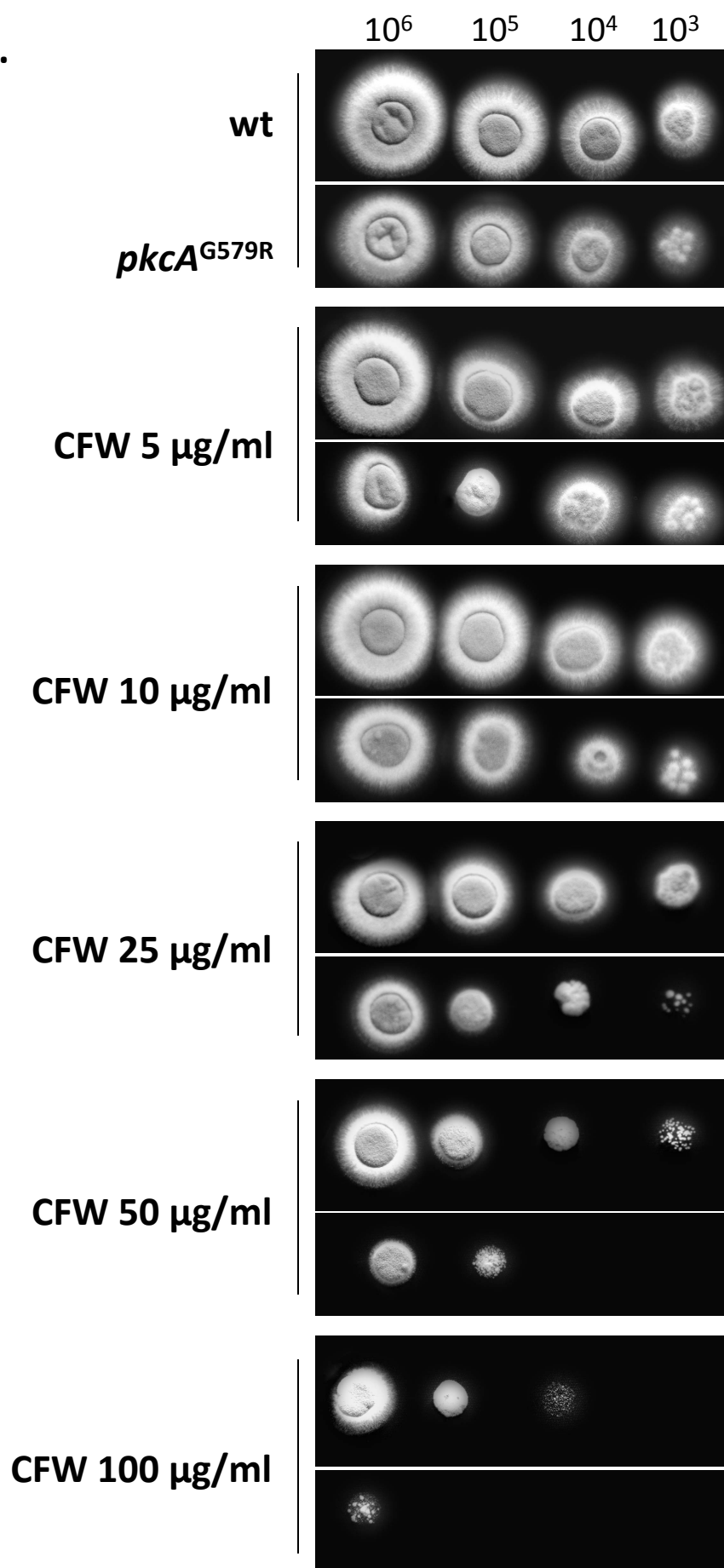

B.

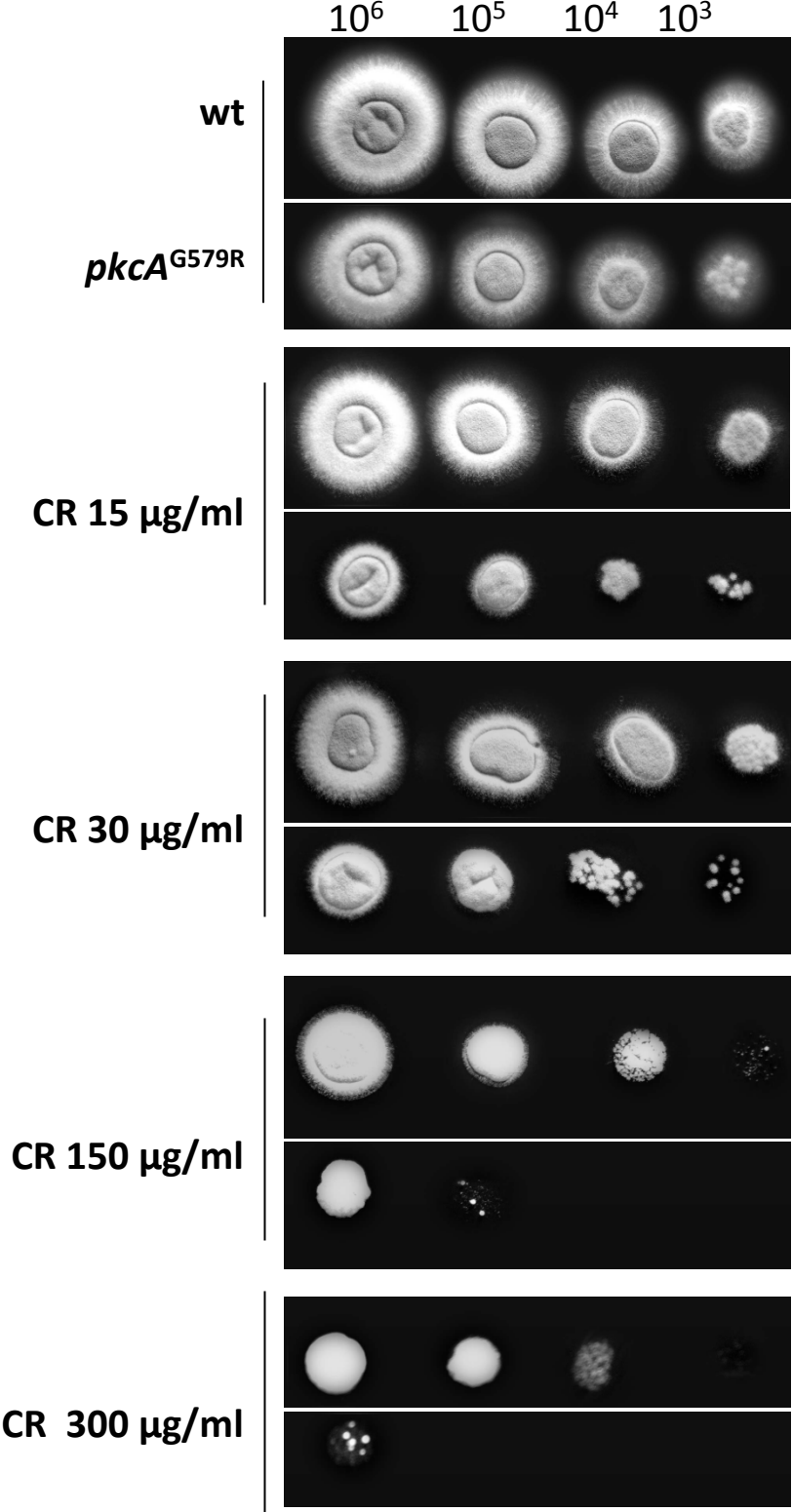

Supplement: S2 Fig — The indicates number of conidia in a 5 μl volume were inoculated in solid YG medium supplemented with CR and CFW. Plates were incubated at 37°C for 3 days. (PDF) [file pone.0135195.s002.pdf]

MM Glucose 1%

MM Glucose 1% + Sorbitol 1.2 M

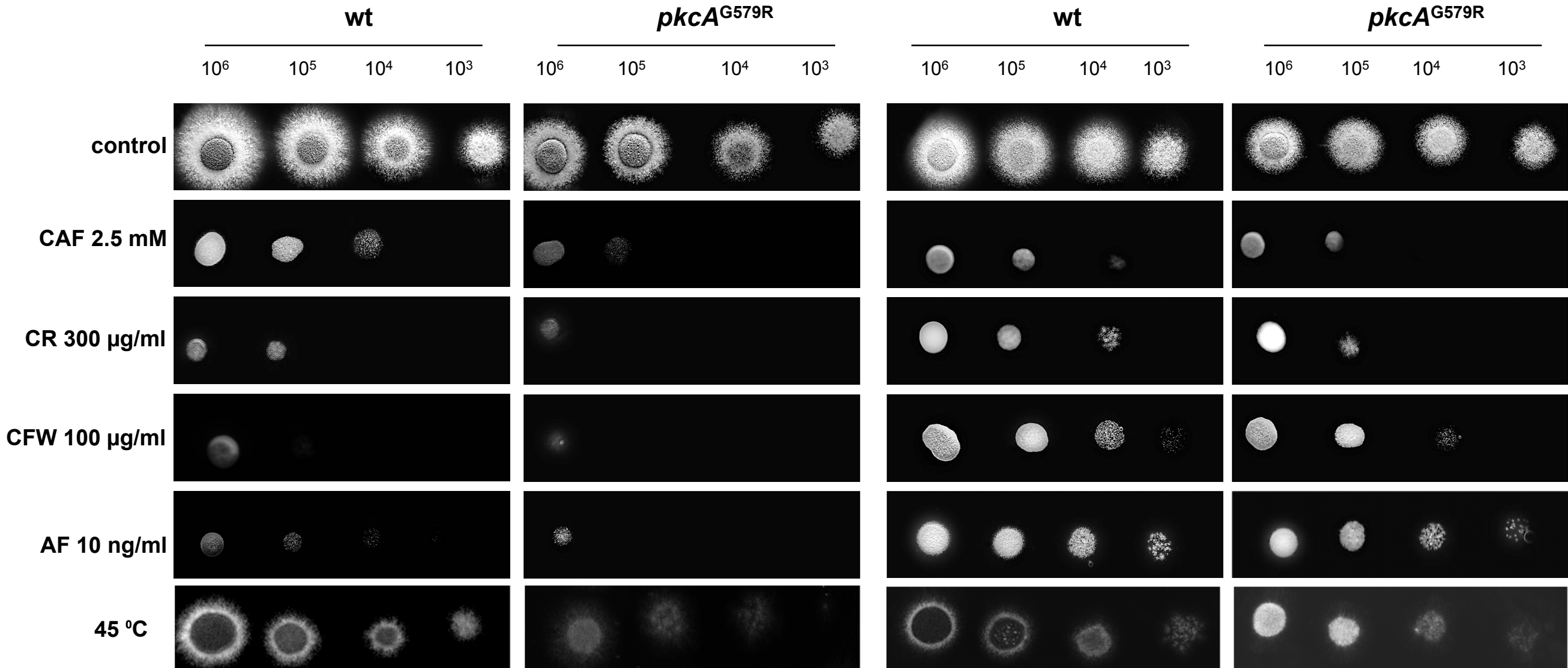

Supplement: S3 Fig — The indicated number of conidia was spotted onto solid MM at 37°C or 45°C, with or without 1.2 M of sorbitol, supplemented with CAF, CR, CFW and AF. The plates were incubated for 2 days at 37°C. (PDF) [file pone.0135195.s003.pdf]

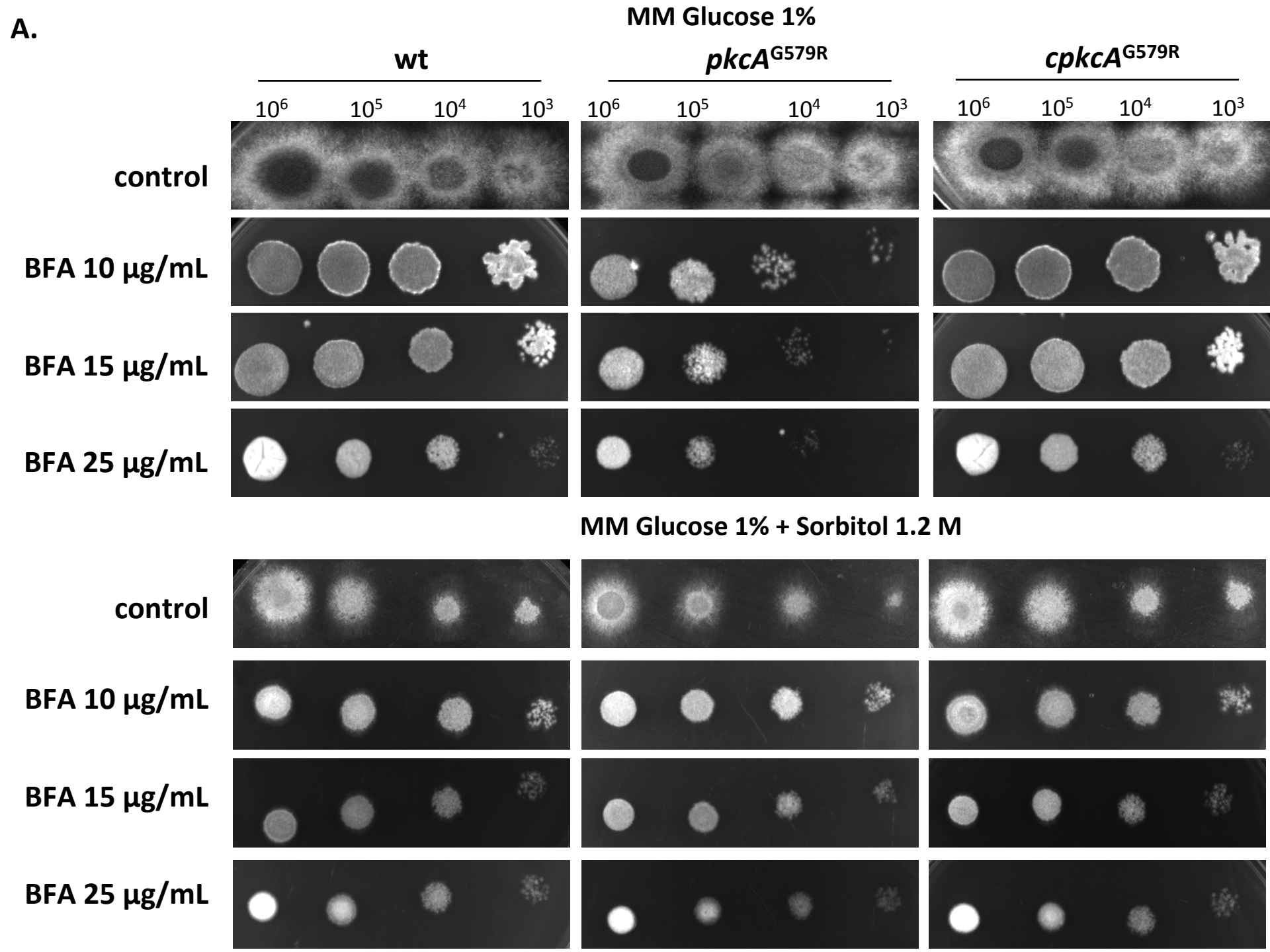

B.

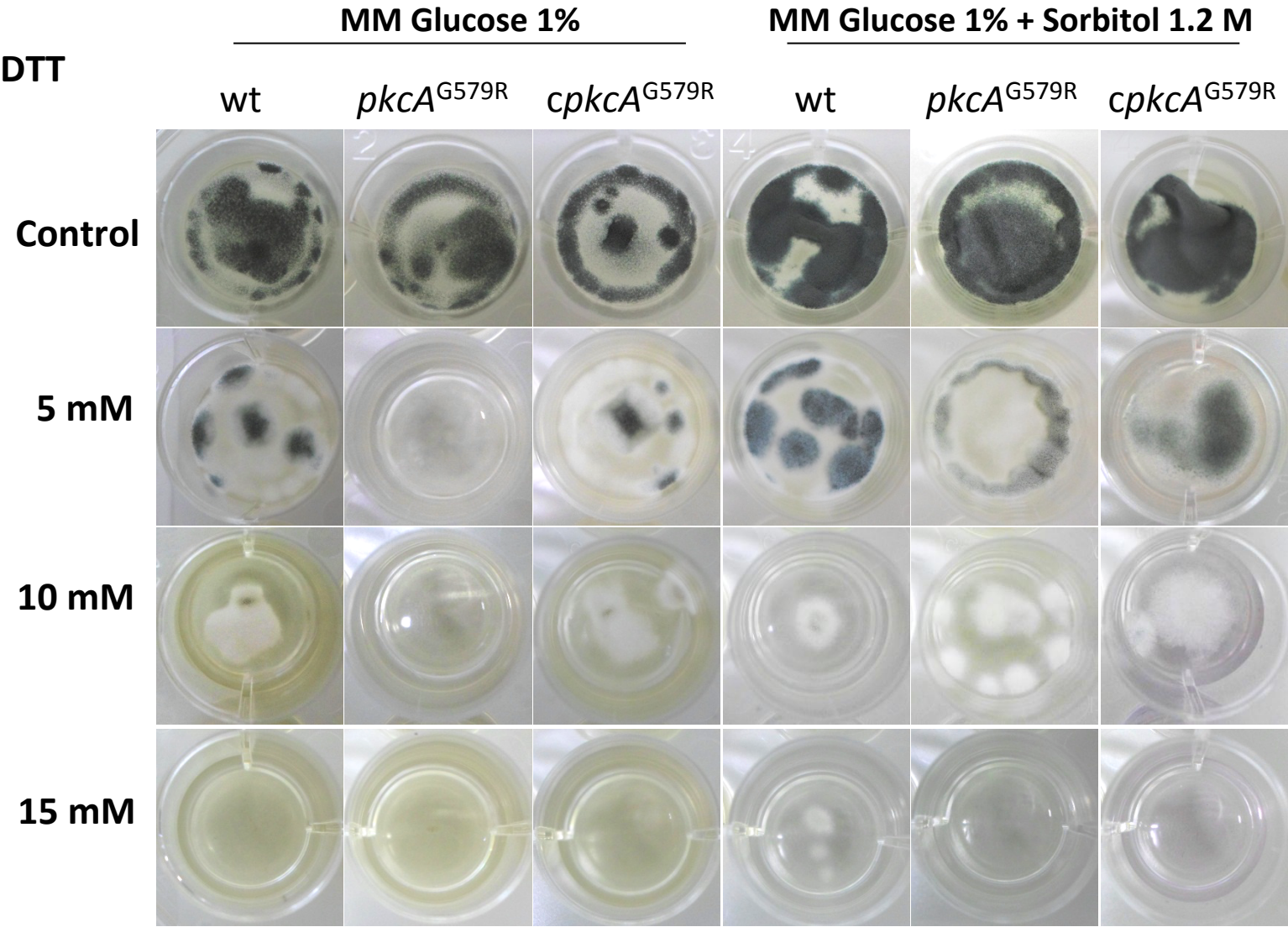

Supplement: S4 Fig — The indicated number of conidia was spotted onto solid MM, with or without 1.2 M of sorbitol, supplemented with brefeldin A (BFA). (B) 1x10^4^ conidia were inoculated in 1 ml of liquid MM in a 24 well plate supplemented with DTT, with or without 1.2 M of sorbitol. The plates were incubated for 3 days at 37°C. (PDF) [file pone.0135195.s004.pdf]
