# Supplementary material for: The Aspergillus fumigatus pkcA G579R Mutant Is Defective in the Activation of the Cell Wall Integrity Pathway but Is Dispensable for Virulence in a Neutropenic Mouse Infection Model
Source: PLoS One. 2015 Aug 21;10(8):e0135195. doi: 10.1371/journal.pone.0135195 (PMC4546635; doi:10.1371/journal.pone.0135195)
Supplement: S1 Table — (PDF) [file pone.0135195.s005.pdf]

Supplemental Table 1: Primers used in this study for mutant construction

Primer name*	Sequence
pkcA START SC	5’-gtaacgccagggttttcccagtcacgacgATGGACGGGGACGACCT-3’
pkcA 2033 REV	5’-GGGACAAGGTGAGTACATTGAG-3’
pkcA GC FW	5’-TTGTCATGCTCAATGTACTCACCTTGTCCCTGACTTTTGT**CGC**ATGTCCA-3’
pkcA 4120 REV	5’-GCATCAGTGCCTCCTCTCAGACAGAATTCCACGAATGAGGAAGACAGTGACC-3’
pyrG FW	5’-GGAATTCTGTCTGAGAGGAGGC-3’
pyrG REV	5’-GATATCGAATTCGCCTCAAAC-3’
pkcA 4145 3F	5’-AAGAGCATTGTTTGAGGCGAATTCGATATCTTTAAGAATATTTAGGGCTAATCGC-3’
pkcA 3R	5’-gcggataacaatttcacacaggaaacagcTCGTCATTTGTATTACCTGCCA-3’
cpkcA FW	5’-TACAACATACCTGGCTGGATG-3’
cpkcA REV	5’-GAGAGGATAGGGAAGATTCTGAAT-3’
Afu5g11970 5F	5’-gtaacgccagggttttcccagtcacgacgTGGGGAAAAGCTTGCAGTT-3’
Afu5g11970 5R	5’-GCCTCCTCTCAGACAGAATTCCTGGTCCGAAGACGAGACAG-3’
Afu5g11970 3F	5’- GTTTGAGGCGAATTCGATATCGTTACTCGGTGTTGATTGAGAATT-3’
Afu5g11970 3R	5’-gcggataacaatttcacacaggaaacagcTCGTCATTTGTATTACCTGCCA-3’

Small letters indicate homology to the pRS426 flanking sequence for the *in vivo* recombination in *S. cerevisiae* (Malavazi *et al.*, 2012).
Underlined letters indicate homology to a fragment in the cassette
* For primers location refer to S1 Fig.

References

Malavazi, I. and Goldman, G.H., (2012) Gene disruption in *Aspergillus fumigatus* using a PCR-based strategy and in vivo recombination in yeast. *Methods Mol Biol* **845**: 99-118.
