# Supplementary material for: The Aspergillus fumigatus pkcA G579R Mutant Is Defective in the Activation of the Cell Wall Integrity Pathway but Is Dispensable for Virulence in a Neutropenic Mouse Infection Model
Source: PLoS One. 2015 Aug 21;10(8):e0135195. doi: 10.1371/journal.pone.0135195 (PMC4546635; doi:10.1371/journal.pone.0135195)
Supplement: S2 Table — (PDF) [file pone.0135195.s006.pdf]

Supplemental Table 2: Real-time PCR primers used in this study

Gene	Systematic name	Primer name	Sequence
pkcA	Afu5g11970	pkcA 2663 FW<br>pkcA 2783 REV	5'-CCGAAGTTCTGTTGGCTCTC-3'<br>5'-CAGAGACCGTAATCGGCAAT-3'
mpkA	Afu4g13720	mpkA FW<br>mpkA REV	5'-GGCCATCAAGAAGGTTACCA-3'<br>5'-TGAAATTGTCTGGTCGTGGA-3'
rlmA	Afu3g08520	rlmA FW'<br>rlmA REV	5'-GACGCCGATCTCTGCTCTAC-3<br>5'-GGAGTGGGGAAGGTTAGAGG-3'
fksA	Afu6g12400	fksA FW<br>fksA REV	5'-AAGCAATCGAAGCTCAGGAA-3'<br>5'-ACCAATCCCATAGAGCGAAC-3'
agsA	Afu3g00910	agsA FW<br>agsA REV	5'-CCAACACCTGGAAGATGACC-3'<br>5'-AACACCGACCGATAGAAGGA-3'
agsB	Afu2g11270	agsB FW<br>agsB REV	5'-TCAGGGATTGGGCTGTATGT-3'<br>5'-TAGCACTTGAGAAGCCAGCA-3'
agsC	Afu1g15440	agsC FW<br>agsC REV	5'-TGCAGACCCTGACAAGAGTG-3'<br>5'-GAACAAGGCAATCCAGAACC-3'
gelA	Afu2g01170	gelA FW<br>gelA REV	5'-CACTGGCTACGGTCTTCCTC-3'<br>5'-CATTGTTGCCGCTAATCTCC-3'
gelB	Afu6g11390	gelB FW<br>gelB REV	5'-CAGGAGGAGAACGACTACGG-3'<br>5'-AGGTCTGGGTTGTGTTGGAG-3'
gelC	Afu2g12850	gelC FW<br>gelC REV	5'-GAATGGTGCGGTGACAAGAC-3'<br>5'-TGTTGCAGCCGTATTCAGAG-3'
chsA	Afu2g01870	chsA FW<br>cshA REV	5'-CTGGAGTGTGGCTGGTCTCT-3'<br>5'-GCGTGTGAAAGCAGTATGGA-3'
chsB	Afu4g04180	chsB FW<br>chsB REV	5'-GCTCTCCACTGTCGGTCTCT-3'<br>5'-GGTCGTTGTTGATGGTGTTG-3'
chsC	Afu5g00760	chsC FW<br>chsC REV	5'-TTGCTGCGAGTTTGTATTCC-3'<br>5'-GCCAGTAGGATGCCAAAGAG-3'
chsD	Afu1g12600	chsD FW<br>chsD REV	5'-CAGAACACGATCCGAACAAC-3'<br>5'-GCTTCGCACCCAAGTAGAAC-3'
chsE	Afu2g13440	chsE FW<br>chsE REV	5'-TGGTGTTCGTTGACTTGCTC-3'<br>5'-TCATCCATCCAACCATTTCC-3'
chsF	Afu8g05630	chsF FW<br>chsF REV	5'-AACCTGCTTCTTCTGGGTGA-3'<br>5'-GAGCACGAGTTCCATGAGGT-3'
chsG	Afu3g14420	chsG FW<br>chsG REV	5'-AGGATGAGGGCAAAGAGGTT-3'<br>5'-AAGGCGTTGCTAAAGATCCA-3'
csmB	Afu2g13430	csmB FW<br>csmB REV	5'- ACAATACGCGGCGAATCC-3'<br>5'- GTTATCCCGACTGCCCAAAA -3'
tubA	Afu1g10910	tubA FW<br>tubA REV	5'-TTCCCAACAACATCCAGACC-3'<br>5'-CGACGGAACATAGCAGTGAA-3'
hacA	Afu3g04070	Afu hacA (u-i) FW<br>Afu hacA (u-i) REV	5'-GTCTGGCTGAGTCCTCCG-3'<br>5'-CCAGAGCCGAGGTCAAAA-3'
